# Effectiveness of Smartphone-Based Dyadic Interventions to Increase Physical Activity in Romantic Couples: Microrandomized Trial

**DOI:** 10.2196/67136

**Published:** 2026-01-27

**Authors:** Patrick Stefan Höhener, Robert Tobias, James Martin Allen, Pascal Küng, Urte Scholz

**Affiliations:** 1 Department of Psychology University of Zurich Zurich Switzerland; 2 School of Health & Wellbeing University of Glasgow Glasgow United Kingdom

**Keywords:** health behavior change, just-in-time adaptive interventions, dyadic interventions, romantic couples, physical activity, microrandomized trial, mobile phone

## Abstract

**Background:**

Social exchange processes, such as social support and social control, can promote health behavior change. However, these processes are often neglected when studying health behavior change and designing interventions. Intervening on these social exchange processes using dyadic interventions may provide a promising approach to promote health behaviors.

**Objective:**

This study aimed to investigate the effects of dyadic interventions to increase moderate-to-vigorous physical activity (MVPA) in romantic couples. Furthermore, we explored how the target, type, and timing of the interventions affect their effectiveness.

**Methods:**

In total, 38 romantic couples (mean age 34.01, SD 11.03 y) were recruited through online advertisements and participated in a smartphone-based microrandomized trial over 55 days consisting of control and intervention phases. The fully automated dyadic interventions included a one-time psychoeducation intervention, weekly dyadic and collaborative planning, and dyadic just-in-time adaptive interventions (JITAIs). MVPA was measured through daily diaries and wrist-worn accelerometers. We used multilevel modeling to estimate the effect of the intervention phase and weighted and centered estimation for microrandomized trials to estimate the treatment effects of dyadic and collaborative planning, as well as the dyadic JITAIs.

**Results:**

Participants indicated higher device-based (b=5.88, SE=3.04, *t*_3665_=1.93; *P*=.03) and self-reported (b=8.26, SE=3.88, *t*_3904_=2.13; *P*=.01) MVPA during the intervention phase compared with the control phase. Dyadic and collaborative planning did not increase device-based (b=6.31, SE=5.18; *P*=.12) but only self-reported (b=14.25, SE=5.16; *P*=.005) MVPA. However, the effects of the 2 kinds of planning on self-reported MVPA disappeared when additional covariates were included (b=0.14, SE=3.32; *P*=.48). Furthermore, the dyadic JITAIs targeting both the actor and the partner increased device-based (actor: b=11.17, SE=3.18; *P*<.001; partner: b=7.23, SE=3.60; *P*=.03) and self-reported (actor: b=17.34, SE=3.65; *P*<.001; partner: b=11.82, SE=4.10; *P*<.001) MVPA. However, the effects of the dyadic JITAIs targeting the actor disappeared for self-reported MVPA (b=2.20, SE=3.22; *P*=0.25) when additional covariates were included. Exploratory analyses revealed that different types and timings of dyadic JITAIs were differentially effective.

**Conclusions:**

This study demonstrated the promising effects of dyadic interventions to promote MVPA and highlighted the importance of the target, type, and timing of the interventions. Further research should investigate the mechanisms underlying the effects of dyadic interventions on health behaviors.

**Trial Registration:**

ISRCTN registry ISRCTN15673058; https://www.isrctn.com/ISRCTN15673058

## Introduction

### Background

Regular engagement in physical activity is essential for maintaining overall health and reducing the risk of chronic and noncommunicable diseases, including heart disease, stroke, diabetes, and cancer [1]. The World Health Organization recommends at least 75 to 150 minutes of moderate-to-vigorous physical activity (MVPA) per week. Even though most people know of its importance and intent to engage in MVPA, many struggle to engage in the recommended amount of MVPA [2]. For example, in Switzerland, where the study took place, about 24% of adults do not meet these recommendations [3].

Theoretical and empirical research has been conducted to support people in increasing their MVPA [4]. This research has primarily focused on behavior change on the individual level and neglected social relationships and social processes [5]. However, since people are embedded in social networks, health behavior change often occurs in a social context. These social networks promote interdependence among people, and especially close partners can influence each other’s health behaviors [6,7]. Thus, involving close social relationships in the behavior change process and implementing dyadic interventions provides a promising approach to promoting health behavior change in people with low physical activity [8].

### Dyadic Interventions for Health Behavior Change

Implementing dyadic interventions makes it possible to capitalize on the interpersonal resources of both partners, encouraging mutual support and enhancing investment in the partners’ behavior change, thereby facilitating behavior change [9-11]. The empirical evidence regarding the effectiveness of dyadic interventions is promising but mixed. On the one hand, some studies have shown that dyadic interventions are effective in increasing physical activity [12] and are more effective than control interventions, including equivalent interventions targeting individuals [9]. On the other hand, systematic reviews found mixed evidence for improved physical health [13], and they did not demonstrate superior effectiveness in increasing physical activity compared with comparison groups involving individual or control interventions [11].

Dyadic interventions may be categorized along the continuum of dyadic interventions into different prototypes of dyadic interventions depending on the degree of partner involvement (eg, cross-over and joint interventions) [10]. Cross-over interventions prompt interactions between the individuals by explicitly instructing one dyad member to engage with or clearly refer to the other dyad member, but the intervention is not administered to both. Joint interventions actively involve both dyad members and consider the dyad as a unit, making it impossible to be delivered to only 1 dyad member present. Cross-over and joint interventions may work through different mechanisms and thus may differ in their effectiveness. However, little research has investigated the differences in their effectiveness, calling for a more nuanced examination of these 2 prototypes of dyadic interventions [10].

Dyadic interventions may work by applying different dyadic behavior change techniques (DBCTs) [10] that explicitly elicit various social exchange processes influencing health behavior change [14]. Social exchange processes are interactions between dyad members that affect one or both individuals’ behaviors, emotions, or cognitions [15]. There are various social exchange processes, such as providing social support, exerting social control, or collaboratively establishing goals and plans. Social support refers to providing psychological, material, or informational resources intended to benefit the ability to cope with stressors, solve problems, and pursue life opportunities [16,17]. One common distinction of social support is between emotional and instrumental support. Emotional support concerns the emotional well-being of the recipient [18]. Regarding physical activity, this form of support may include encouragement and comfort, guidance, and help to stay motivated and committed. Instrumental support is defined as providing the recipient with practical help and resources, such as advice or assistance [18]. Regarding physical activity, this form of support could include providing help while exercising or taking over daily chores to free up time for the partner to engage in MVPA. Empirical evidence showed that emotional and instrumental support can be associated with higher levels of physical activity, but there is also substantial heterogeneity in the associations (eg, studies by Kouvonen et al [19], Rackow et al [20], and Scarapicchia et al [21]).

Another social exchange process is social control, which refers to a deliberate and intentional attempt to change what another person thinks, feels, or does toward an outcome desired by the person exerting control [22]. Social control can be categorized into positive or negative social control based on the means used to influence behavior [23]. Positive social control includes strategies such as persuasion, modeling, and discussions. Negative social control includes, for example, coercion, social pressure, elicitation of negative emotions, and withdrawal. These strategies may differ in effectiveness when used to promote health behaviors in the partner. A meta-analysis has found that positive control was associated with better health behaviors, whereas negative control was related to worse health behaviors [22]. Furthermore, negative control relates to increased reactance-related responses and resistance to change, rendering it ineffective in encouraging health behavior change [7]. So far, there is almost no research on the effectiveness of interventions promoting positive social control and reducing negative social control. This gap will be addressed in this study.

While social support and positive social control can enhance health behaviors, poorly executed or nonresponsive support may yield adverse outcomes, including diminished well-being, heightened stress, and decreased performance [24]. Therefore, when deploying dyadic interventions that leverage social exchange processes, it seems crucial for partners to have the knowledge and skills to offer effective support. However, little research has included information on skillful social support in interventions.

Social exchange processes can also play a role in goal setting and action planning. Goal setting refers to defining behaviors or states one wants to accomplish in the future [25]. Action planning refers to linking behaviors to specific cues by specifying when, where, and how to act [26]. Action planning can be augmented by coping planning, a barrier-focused self-regulation strategy in which an association between anticipated barriers and suitable solutions to overcome these barriers is made [26]. Combining action planning with coping planning can have additive and synergistic effects to promote health behaviors [27]. Furthermore, setting goals and planning physical activities with a partner may facilitate adherence to these goals and plans [5,28]. There are 2 forms of planning in the dyadic context. Dyadic planning involves creating plans with a partner on when, where, and how one partner will implement a behavior [29]. Collaborative planning entails creating joint plans with a partner on when, where, and how both partners will engage in a behavior [30]. There is mixed evidence for the effectiveness of dyadic planning in promoting health behaviors. While some studies have found positive associations between dyadic planning and goal progress [31] and plan enactment [32], other studies have not supported its effectiveness [33,34]. However, empirical evidence generally supports the effectiveness of collaborative planning to foster health behaviors [28,35,36].

### Just-in-Time Adaptive Interventions

Technological advancements and the widespread use of devices, such as smartphones and smartwatches, have opened up new possibilities for enhancing the design and implementation of interventions [37]. These technologies allow for tracking health behaviors and context variables, which provide information about the individual’s current state. This information can be integrated with theoretical and empirical knowledge to tailor the intervention content to individual needs and deliver interventions at the right time. Such interventions are known as just-in-time adaptive interventions (JITAIs) [38]. A meta-analytical review found moderate to large effects of JITAIs compared with waitlist-control conditions and non-JITAI treatments to improve health behaviors [39]. However, regarding physical activity and sedentary behavior, the evidence supporting the effectiveness of JITAIs is mixed [37]. However, so far, only JITAIs aimed at individuals have been implemented. Combining DBCTs with JITAIs and intervening on a dyadic level may provide a promising approach to increasing their effectiveness in promoting MVPA.

### This Study

This study aimed to examine the effectiveness of dyadic interventions in promoting MVPA in romantic couples. Specifically, we investigated the effectiveness of dyadic interventions containing various DBCTs that elicit social exchange processes for increasing MVPA in romantic couples. We implemented an intensive-longitudinal study over 55 days consisting of phases without dyadic interventions (ie, control phase) and with dyadic interventions, including a one-time psychoeducation intervention on skilled support, weekly planning interventions, and dyadic JITAIs (ie, intervention phase). In the psychoeducation intervention on skilled support, participants learned how to support their partner appropriately to increase their MVPA. In the planning interventions, the participants set a goal and planned the physical activities for the upcoming week. Finally, there were dyadic JITAIs targeting various social exchange processes to increase MVPA.

To investigate the effectiveness of the dyadic interventions, we compared the MVPA during the intervention phase with the control phase. To isolate the effects of the different dyadic interventions, we investigated the effectiveness of the planning interventions and the dyadic JITAIs separately. Note that we did not examine the effect of the skilled support intervention since this was a one-time intervention taking place right after the baseline week and is, therefore, highly correlated with the intervention phase.

We proposed the following hypotheses, all of which were preregistered on Open Science Framework (OSF):

- Hypothesis 1: Couples show higher MVPA (device-based and self-reported) during the intervention phase than during the control phase.
- Hypothesis 2: On days for which participants had planned to be physically active during the planning intervention, they engage in more MVPA (device-based and self-reported) compared with days for which they had not planned any physical activity.
- Hypothesis 3a: Participants indicate higher MVPA (device-based and self-reported) on days when a dyadic JITAI targeted the actor’s MVPA compared with days without a dyadic JITAI targeting the actor.
- Hypothesis 3b: Participants indicate higher MVPA (device-based and self-reported) on days when a dyadic JITAI targeted the partner compared with days without a dyadic JITAI targeting the partner.

Additionally, we conducted 3 exploratory analyses. First, we explored the effect of different types of dyadic JITAIs by differentiating between cross-over and joint interventions. Second, we explored the effectiveness of the dyadic JITAIs depending on their timing (ie, when they were sent). There were dyadic JITAIs sent before the planning intervention, before a planned activity, and in the evening. These 3 timings target different aspects of the behavior change process, which may vary in their effectiveness. Finally, we explored how long the effects of the dyadic JITAIs last. Dyadic JITAIs may make lasting changes in the interaction patterns between the partners, allowing their effects to be maintained over time. Thus, these changes in the social exchange processes may go beyond the specific day targeted by the dyadic JITAIs, facilitating engagement in MVPA at a later occasion.

## Methods

### Funding and Preregistration

This study is part of the “Time and Ties: Dynamic modelling of temporal patterns in dyadic health behaviour change” project funded by the Swiss National Science Foundation (grant 10001C_197471 / 1). A comprehensive description of the project can be found on the OSF page of the Time and Ties project [40]. The preregistration, including hypotheses, inclusion and exclusion criteria, and planned primary analyses, can be found on the OSF page of the study [41]. The study differs from the preregistration in that we conducted the dyadic analyses for indistinguishable instead of distinguishable dyads. This decision was made to be inclusive of same-gender couples. The completed CONSORT-EHEALTH (Consolidated Standards of Reporting Trials of Electronic and Mobile Health Applications and Online Telehealth) checklist can be found in Multimedia Appendix 1.

### Ethical Considerations

The study received approval from the Ethics Committee of the Faculty of Arts and Social Sciences of the University of Zurich (approval 21.9.11). All participants provided online informed consent using a checkbox before enrolling in the study and were free to withdraw at any time (the informed consent is provided in Multimedia Appendix 2). All collected data are stored in anonymized form on the server of the University of Zurich and are only accessible to the project team. All participants received 150 Swiss Francs (approximately US $189) for their complete participation.

### Participants

Data collection took place from June to October 2022. Romantic couples were recruited from the Swiss population through online advertisements on Facebook (Meta) and Instagram (Meta). Inclusion criteria were (1) both partners must be at least 18 years old, (2) they must be in a romantic relationship with each other and live together, (3) both partners must be physically active for less than 150 minutes per week, (4) both partners must have the intention to be more physically active, (5) both partners must not have severe health conditions preventing physical activity, (6) both partners must have a smartphone that they use regularly and have sufficient literacy to operate it independently, and (7) both partners must speak German fluently.

Descriptive statistics of the sample are presented in Table 1.

**Table 1:** Descriptive statistics of the sample characteristics (76 participants; 38 couples).

Variable		Value
**Gender, n (%)**		
	Women	39 (51)
	Men	37 (49)
**Education, n (%)**		
	Vocational education	8 (11)
	High school diploma	24 (32)
	Bachelor’s degree	23 (30)
	Master’s degree	21 (28)
Married, n (%)		26 (34)
Having children, n (%)		21 (28)
Age (y), mean (SD; range)		34.01 (11.03; 19-60)
BMI (kg/m^2^), mean (SD; range)		24.94 (4.11; 16.37-33.95)
Relationship duration (y), mean (SD; range)		9.23 (9.09; 0.58-36.00)
Cohabitation duration (y), mean (SD; range)		7.53 (9.20; 0.25-33.00)
Age of children (n=21; y), mean (SD; range)		16.16 (9.24; 4-32)

### Study Design

We conducted an intensive-longitudinal microrandomized trial over 55 days. This study was the first empirical study within the larger Time and Ties project and aimed to inform a computational modeling approach on dyadic interventions as well as to test and improve the smartphone-based data gathering and intervention provision. Thus, the study was not powered for advanced statistical analyses of dyadic intervention effects. From this perspective, it can be considered a pilot trial. An overview of the larger Time and Ties project, including this study, can be found on OSF. The study was smartphone-based, using a self-developed app in collaboration with a company specializing in app development (Multimedia Appendix 3). Participants read the information about the study and provided written informed consent before the start of the study. They were informed that various interventions would be implemented during the study. After registration, the participants received a link to download and sign in to the app. Throughout the study, participants completed daily diaries and wore wrist-worn accelerometers. The study consisted of phases without dyadic interventions (ie, control phase) and with dyadic interventions, including a 1-time psychoeducation intervention about skilled support, weekly planning interventions, and dyadic JITAIs (ie, intervention phase). Random allocation was implemented using a computer program that assigned couples to the intervention conditions characterized with different intervention phases, while ensuring equal sample sizes across groups (ie, blocked randomization). Each group started with a 1-week control phase to establish a baseline. Afterwards, group A received a 7-week intervention phase. Group B received a 3-week intervention phase followed by a 4-week control phase. Group C received a 4-week control phase followed by a 3-week intervention phase at the end of the study (Figure S1 in Multimedia Appendix 4). During the intervention phase, various dyadic interventions were implemented on designated days, but there were also randomly assigned control days without any interventions. Participants were aware that there were interventions during the study, but they did not know which intervention conditions there were. At the end of the study, participants completed a questionnaire similar to the baseline questionnaire, where they also had the opportunity to provide qualitative feedback. All questionnaires and interventions were sent using a smartphone app designed for this study.

### Interventions

#### Skilled Support Intervention

The psychoeducation intervention about the principle of skilled support took place on the first Saturday after the 1-week control phase. During this intervention, couples learned about different types of social support and social control, the importance of the right timing and equity of support, and how to support each other effectively to help engage in MVPA (DBCTs: “one partner receives education for supporting the other partner,” which was applied by both partners) [24]. Furthermore, partners discussed and reported how and when they would like to be supported and how they communicate about social support and social control (DBCTs: “one partner identifies preferred support strategies for the other partner,” “one partner gives feedback on support provision of the other partner,” and “one partner practices communication skills for health behavior of the other partner;” all of which were applied by both partners).

#### Planning Interventions

The planning interventions occurred each Sunday during the intervention phase and consisted of 3 parts. First, the couple set their weekly goal by indicating the desired duration of MVPA they aimed to achieve in the upcoming week (DBCTs: “the couple sets a goal for the couple” and “the couple commits to a goal of the couple”) [42]. Second, they planned physical activities for the upcoming week. Thereby, they could either plan dyadically (ie, plan together activities they want to do individually) [29] or collaboratively (ie, plan together activities they want to do together) [30]. They planned the activity, timing, location, duration, and whether they would participate individually or together in the planned activity (DBCTs: “the couple plans for one partner” and “the couple plans for the couple”). Third, they engaged in coping planning by anticipating potential barriers that may prevent them from engaging in the planned activities and discussing solutions to overcome them (DBCTs: “the couple creates a coping plan for one partner” and “the couple creates a coping plan for the couple”) [26].

#### Dyadic JITAIs

The dyadic JITAIs were designed in accordance with the recommendations proposed by Nahum-Shani et al [38]. The JITAIs targeted various social exchange processes within the couple that were hypothesized to promote engagement in MVPA (a complete list of all the DBCTs that were implemented can be found on OSF). Note that the target of the JITAI is not necessarily the person who receives the JITAI. For example, a JITAI prompting to provide emotional support to engage in MVPA may be sent to one partner (ie, execution level) but targeting the other (ie, target level) [10]. Depending on the content of the JITAIs, they could target either one or both partners’ MVPA. However, due to the interdependence between the partners, the effects may spill over to the nontargeted partner [43]. Thus, this study included JITAIs targeting the actor’s MVPA and JITAIs targeting the partner’s MVPA. Note that both JITAIs targeting the actor and the partner may be present on the same day (ie, through joint or 2 cross-over interventions targeting different partners).

There were 2 types of dyadic JITAIs: cross-over and joint JITAIs [10]. Cross-over JITAIs were sent to one partner and included instructions to interact with the other partner (eg, provide emotional support to the partner). Joint JITAIs were sent to both partners and included instructions to both partners to actively interact with each other (eg, jointly create a list of advantages of physical activity). The JITAIs were sent to the participants at a specified time, hypothesized to be the most appropriate for the JITAI content. There were three timings when the JITAIs could be triggered: (1) before the planning intervention, (2) before a planned activity, and (3) in the evening. The dyadic JITAIs before the planning intervention targeted the social exchange processes during the planning; those before the planned activity targeted the social exchange processes (eg, social support and social control) before and during the activity; and those in the evening targeted the social exchange processes during the reflection about the engagement in MVPA. Depending on the timing of the JITAIs, the expected effect might not manifest on the same day the couple received the dyadic JITAI but may be on a subsequent day they intended to engage in MVPA (Figures S2-S4 in Multimedia Appendix 4).

Each day, a randomization process either selected a JITAI or not, the latter serving as a control condition. Multimedia Appendix 4 provides a description of the dyadic JITAIs and the selection process. All interventions were stand-alone interventions and were triggered automatically without human involvement.

### Measures

#### Intervention Variables

This study comprised several variables for the different intervention components. First, the study was categorized into the control phase (ie, phase without any dyadic interventions, serving as the reference category) and intervention phase (ie, phase with dyadic interventions). Second, planning was categorized into days without planned physical activities (coded as 0) and days for which the couples had planned physical activities during the planning intervention (coded as 1). Third, regarding the dyadic JITAIs, we included various dummy variables to describe whether a specified JITAI targeted the MVPA that day or did not target the day. We defined two variables for JITAIs targeting the actor and the partner: (1) the dyadic JITAI for the actor compared days on which a dyadic JITAI targeted the actor’s MVPA (coded as 1) with days without dyadic JITAI targeting the actor’s MVPA (coded as 0), and (2) the dyadic JITAI for the partner compared days on which a dyadic JITAI targeted the partner’s MVPA (coded as 1) with days without dyadic JITAI targeting the partner’s MVPA (coded as 0). For the exploratory analyses, we created two variables representing the types of JITAI: (1) cross-over JITAI (coded as 1) versus no cross-over JITAI (coded as 0) targeting the MVPA on that day, and (2) joint JITAI (coded as 1) versus no joint JITAI (coded as 0) targeting the MVPA on that day. Joint JITAIs were coded as targeting both the actor’s and partner’s MVPA. Moreover, we created three variables representing the timing of the JITAI: (1) JITAI before the planning intervention (coded as 1) versus no JITAI before the planning intervention (coded as 0) targeting the MVPA that day, (2) JITAI before the activity (coded as 1) versus no JITAI before the activity (coded as 0) targeting the MVPA that day, and (3) JITAI in the evening (coded as 1) versus no JITAI in the evening (coded as 0) targeting the MVPA that day. Finally, we computed three lagged terms of the JITAI variables to describe how many occasions ago the JITAI targeted the MVPA: (1) JITAI targeting the MVPA 1 occasion before (coded as 1) compared with no JITAI targeting the MVPA one occasion before (coded as 0), (2) JITAI targeting the MVPA 2 occasions before (coded as 1) compared with no JITAI targeting the MVPA 2 occasions before (coded as 0), and (3) JITAI targeting the MVPA 3 occasions before (coded as 1) compared with no JITAI targeting the MVPA 3 occasions before (coded as 0).

#### Physical Activity

Physical activity was assessed using both device-based and self-reported methods. The device-based physical activity in minutes of MVPA per day was measured using wrist-worn accelerometers (ActiGraph CentrePoint Insight Watches [Ametris]). The acceleration is measured on 3 axes from which a single vector magnitude count is calculated. Values above 2690 counts per minute were classified as MVPA [44]. The device-based MVPA was filtered for wear time of at least 10 hours per day, according to Choi et al [45], and for awake time, according to the algorithm proposed by Tracy et al [46]. Overall, 11.3% (n=474) of the responses on device-based MVPA are missing after excluding days with low wear-compliance. The self-reported MVPA was measured by adding up the 2 questions capturing the MVPA they did alone (“How many minutes did you spend today alone doing moderate-to-vigorous physical activity?”) and with their partner (“How many minutes did you spend today together with your partner doing moderate-to-vigorous physical activity?”). Overall, 5.6% (n=236) of the responses for self-reported MVPA are missing.

#### Covariates

We assessed the intervention group with 2 time-invariant dummy covariates (group A serving as the reference category) to address differences due to different sequences in the intervention and control phases. Furthermore, we assessed various time-variant covariates. We included the wear time of the wrist-worn accelerometer (minutes per day) because increased wear time increases the potential duration of MVPA that can be recorded. Moreover, we included the time (per 7 days) to address potential time trends [47]. Furthermore, we included dummy variables for the weekend (weekday serving as the reference category) to address differences in the behavior between weekdays and weekends, and skilled support intervention (before the intervention serving as the reference category) to address the potential effects of this intervention on the MVPA. Finally, we assessed the barriers and facilitating factors. Barriers and facilitating factors were assessed with the item “What has made your physical activity easier or more difficult today?” on a bipolar scale from −5 to 5, on which the participants responded to 8 influences that hindered or facilitated engagement in physical activity (eg, “hindering conditions for physical activity” to “beneficial conditions for physical activity”). The scores of the barriers and facilitating factors were summed up into 2 separate scores, indicating the total barriers and facilitating factors today. We included the barriers and facilitating factors to control the internal and external influences that may change the probability or duration of the engagement in MVPA and to test if the interventions effectively increased MVPA over and above these factors [48].

### Data Analysis

To estimate the treatment effect of the intervention phase, we calculated multilevel models for intensive longitudinal data and indistinguishable dyads [49]. This approach can be used to analyze dyadic data by accounting for the nonindependence of observations within dyads. We included the intervention phase as the predictor variable and the device-based or self-reported MVPA as the outcome variables.

We used weighted and centered least-squares estimation for microrandomized trials to estimate the treatment effects of the planning interventions and the dyadic JITAIs [50]. This approach addresses the potential biases of time-varying treatment effects and provides consistent causal effects in microrandomized trials. We extended this approach to dyadic data by considering the dyads as the units of analysis. To be inclusive of same-gender couples in our study, we treated the partners as indistinguishable rather than distinguishable based on gender [49]. We included the planning or the JITAI targeting the actor and partner as predictor variables, respectively. We ran separate models with device-based and self-reported MVPA as outcome variables. In all models, complete case analyses were conducted. Thus, days with missing data were excluded from the data analyses. We included time as a covariate in all models [47]. Additionally, in analyses of device-based MVPA, we included the wear time of the wrist-worn accelerometer as an additional covariate.

Furthermore, we conducted sensitivity analyses to account for potential influences of covariates. In these sensitivity analyses, we additionally included variables indicating the intervention group, weekend, skilled support intervention, barriers, and facilitating factors as covariates. All covariates were grand mean-centered. All analyses were complete-case analyses.

We ran 3 separate models for the exploratory analyses. In these exploratory analyses, we included the dyadic JITAI type (ie, cross-over and joint), the JITAI timing (ie, before planning, before the activity, and evening), and the lagged terms of the JITAI as dummy variables.

### Software

The analyses were conducted with R in RStudio [51,52]. We used the nlme package (v3.1-157) to estimate the effect of the intervention phase [53] and the xgeepack package (v1.3.9) to estimate the causal treatment effects of dyadic and collaborative planning and dyadic JITAIs [50,54,55]. All R codes are provided on OSF.

## Results

### Overview

**Figure 1:**
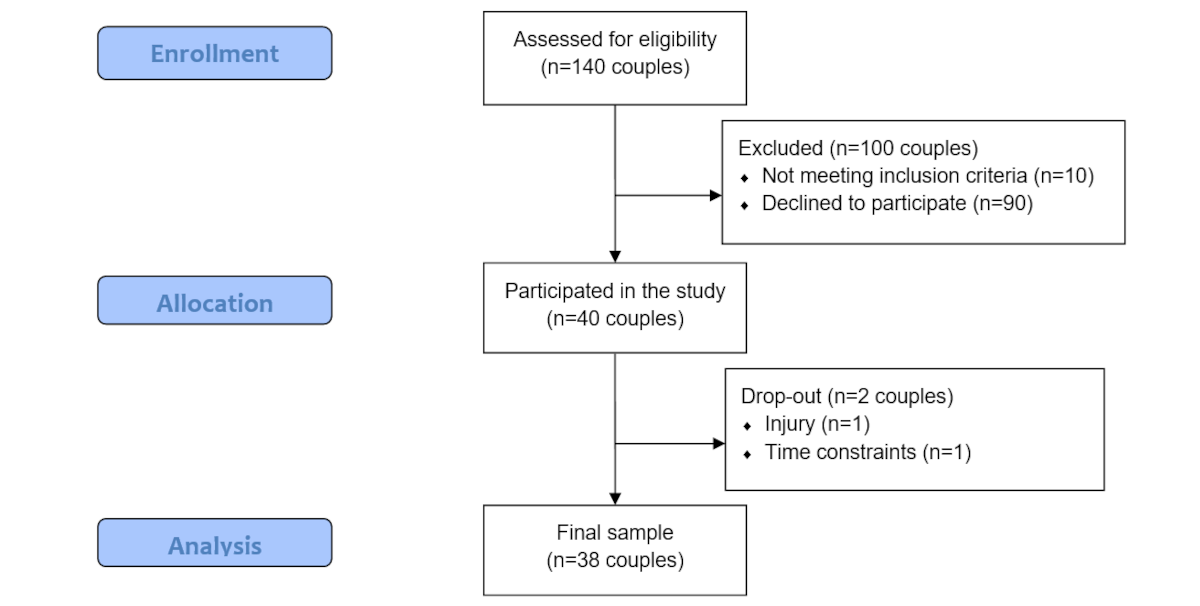
CONSORT (Consolidated Standards of Reporting Trials) flow diagram.

Initially, 140 couples signed up for the study. However, 100 of them either chose not to participate or were excluded during screening. Thus, a total of 40 couples (80 participants) participated in the study, which accounted for an expected attrition rate of 20%. The recruitment stopped when the planned sample size was reached. Furthermore, 1 couple dropped out of the study due to an injury of 1 partner, and 1 couple discontinued the study because of time issues, leaving a total sample of 38 couples (37 mixed-gender and 1 same-gender couple). The CONSORT (Consolidated Standards of Reporting Trials) flow diagram is shown in Figure 1. The mean duration of MVPA was 116.64 (SD 35.40) minutes per day for device-based MVPA and 30.24 (SD 22.19) minutes per day for self-reported MVPA. There is a moderate to high correlation between the device-based and self-reported MVPA, with *r_b_*=0.57 (*P*<.001) at the between-person and *r_w_*=0.46 (*P*<.001) at the within-person level.

Tables 2 and 3 present the effects of the intervention phase on device-based and self-reported MVPA, expressed as the difference in minutes between days during the intervention phase compared with days during the control phase. In line with hypothesis 1, the results indicated that during the intervention phase, the couples had significantly higher device-based and self-reported MVPA than during the control phase. Furthermore, the intercepts and slopes showed significant variability, indicating heterogeneity in the mean levels of MVPA and the effects of the intervention phases on the MVPA between the couples.

**Table 2:** Effects of the intervention phase on device-based moderate-to-vigorous physical activity.

Effect^a^		Estimate, b (SE)	90% CI	*t* test (*df*) or *z* score	*P* value^b^
**Fixed effects**					
	Intercept	106.50 (4.39)	99.29 to 113.72	24.28 (3665)^c^	<.001
	Intervention phase^d^	5.88 (3.04)	0.88 to 10.89	1.93 (3665)^c^	.03
	Time^e^	−1.05 (0.85)	−2.44 to 0.34	−1.24 (3665)^c^	.22
	Wear time^f^	0.15 (0.01)	0.13 to 0.17	13.04 (3665)^c^	<.001
**Random effects**					
	Intercept	24.30 (3.30)	19.48 to 30.33	7.37^g^	<.001
	Intervention phase	8.59 (3.60)	4.51 to 16.36	2.39^g^	.02
	Time	4.21 (0.83)	3.06 to 5.79	5.06^g^	<.001
	Wear time	0.06 (0.01)	0.05 to 0.08	5.69^g^	<.001
	Residuals	49.89 (0.65)	48.83 to 50.97	76.53^g^	<.001
	Autocorrelation	0.27 (0.02)	0.24 to 0.30	15.67^g^	<.001

^a^Number of couples=38; number of days=55; number of cases in the analysis=3706.

^b^All *P* values are 2-tailed except those of the hypothesized effect of the intervention phase, where 1-tailed *P* values are used.

^c^*t* test.

^d^Intervention phase (coded as 1) included all days during the period when the couple received dyadic interventions and was compared with the control phase (coded as 0).

^e^Time was grand mean-centered per 7 days.

^f^Wear time of the accelerometers in minutes was grand mean-centered.

^g^*z* score.

**Table 3:** Effects of the intervention phase on self-reported moderate-to-vigorous physical activity.

Effects^a^				Estimate, b (SE)		90% CI		*t* test (*df*) or *z* score		*P* value^b^
**Fixed effects**										
	Intercept			26.65 (3.17)		21.44 to 31.86		8.41 (3904)^c^		<.001
	Intervention phase^d^			8.26 (3.88)		1.88 to 14.63		2.13 (3904)^c^		.02
	Time^e^			−2.28 (0.95)		−3.84 to −0.71		−2.40 (3904)^c^		.02
**Random effects**										
	**Level-2**									
		Intercept	16.95 (2.50)		13.33 to 21.54		6.79^f^		<.001	
		Intervention phase	19.01 (3.50)		14.11 to 25.62		5.43^f^		<.001	
		Time	5.20 (0.79)		4.06 to 6.67		6.55^f^		<.001	
	**Level-1**									
		Residuals	49.14 (0.57)		48.22 to 50.08		86.93^f^		<.001	
		Autocorrelation	0.03 (0.02)		0.00 to 0.06		1.74^f^		.08	

^a^Number of couples=38; number of days=55; number of cases in the analysis=3994.

^b^All *P* values are 2-tailed except those of the hypothesized effect of the intervention phase, where 1-tailed *P* values are used.

^c^*t* test.

^d^Intervention phase (coded as 1) included all days during the period when the couple received dyadic interventions and was compared to the control phase (coded as 0).

^e^Time was grand mean-centered per 7 days.

^f^*z* score.

The average treatment effects of the planning interventions on device-based and self-reported MVPA in minutes are presented in Table 4. The results indicate that on days the participants planned to be physically active during the planning interventions, they indicated higher self-reported but not device-based MVPA. Thus, hypothesis 2 is only supported for self-reported but not device-based MVPA.

**Table 4:** Effects of the dyadic and collaborative planning interventions on device-based and self-reported moderate-to-vigorous physical activity.

Parameter^a^	Device-based MVPA^b,c^				Self-reported MVPA^d^		
	Estimate, b (SE)	90% CI	*P* value^e^	Estimate, b (SE)		90% CI	*P* value^e^
Intercept	107.59 (3.79)	101.19 to 114.00	<.001	30.27 (3.51)		24.34 to 36.21	<.001
Planning^f^	6.31 (5.18)	−2.45 to 15.06	.12	14.25 (5.16)		5.53 to 22.98	.005
Time^g^	−1.59 (0.87)	−3.06 to −0.12	.08	−2.27 (0.85)		−3.71 to −0.83	.01
Wear time^h^	0.16 (0.01)	0.13 to 0.18	<.001	—^i^		—	—

^a^Number of couples=38; number of days=55.

^b^MVPA: moderate-to-vigorous physical activity.

^c^Number of cases in the analysis for device-based moderate-to-vigorous physical activity=3706.

^d^Number of cases in the analysis for self-reported moderate-to-vigorous physical activity=3944.

^e^All *P* values are 2-tailed except those of the hypothesized effects of the planning interventions, where 1-tailed *P* values are used.

^f^Days with planned physical activities (coded as 1) were compared with days without any planned physical activities (coded as 0).

^g^Time was grand mean-centered per 7 days.

^h^Wear time of the accelerometers in minutes was grand mean-centered.

^i^Not applicable.

The average treatment effects of the dyadic JITAIs on device-based and self-reported MVPA in minutes are presented in Table 5. In line with hypothesis 3, the results indicated that both the dyadic JITAIs targeting the actor as well as the partner significantly increased the device-based and self-reported MVPA.

**Table 5:** Effects of the dyadic just-in-time adaptive interventions on device-based and self-reported moderate-to-vigorous physical activity.

Parameter^a^	Device-based MVPA^b,c^				Self-reported MVPA^d^		
	Estimate, b (SE)	90% CI	*P* value^e^	Estimate, b (SE)		90% CI	*P* value^e^
Intercept	107.62 (3.73)	101.31 to 113.93	<.001	30.17 (3.47)		24.30 to 36.04	<.001
Dyadic JITAI_Actor_^f,g^	11.17 (3.18)	5.79 to 16.55	<.001	17.34 (3.65)		11.17 to 23.51	<.001
Dyadic JITAI_Partner_^h^	7.23 (3.60)	1.14 to 13.33	.03	11.82 (4.10)		4.89 to 18.75	.003
Time^i^	−1.64 (0.79)	−2.97 to −0.30	.046	−2.17 (0.73)		−3.41 to −0.93	.006
Wear time^j^	0.16 (0.01)	0.13 to 0.18	<.001	—^k^		—	—

^a^Number of couples=38; number of days=55.

^b^MVPA: moderate-to-vigorous physical activity.

^c^Number of cases in the analysis for device-based moderate-to-vigorous physical activity=3706.

^d^Number of cases in the analysis for self-reported moderate-to-vigorous physical activity=3944.

^e^All *P* values are 2-tailed except those of the effects of the hypothesized dyadic just-in-time adaptive interventions, where 1-tailed *P* values are used.

^f^JITAI: just-in-time adaptive intervention.

^g^Days on which a dyadic just-in-time adaptive intervention targeted the actor’s moderate-to-vigorous physical activity (coded as 1) were compared with days without dyadic just-in-time adaptive interventions targeting the actor’s moderate-to-vigorous physical activity (coded as 0).

^h^Days on which a dyadic just-in-time adaptive intervention targeted the partner’s moderate-to-vigorous physical activity (coded as 1) were compared with days without dyadic just-in-time adaptive interventions targeting the partner’s moderate-to-vigorous physical activity (coded as 0).

^i^Time was grand mean-centered per 7 days.

^j^Wear time of the accelerometers in minutes was grand mean-centered.

^k^Not applicable.

### Sensitivity Analyses

Results of the sensitivity analyses, including the effect of the intervention phase, the average treatment effects of the planning, and the dyadic JITAIs, are reported in Tables S1-S3 in Multimedia Appendix 5. The pattern of the effects of the intervention phase on the device-based and self-reported MVPA remained the same after controlling for various covariates, suggesting the robustness of the results. However, the average treatment effect of the planning intervention on self-reported MVPA disappeared when controlling for covariates. Similarly, the average treatment effect of the dyadic JITAIs targeting the actor on the self-reported MVPA was no longer significant when adding the covariates. We further explored which covariates were responsible for these different results compared with the main analyses. Excluding the barriers and facilitating factors from the sensitivity analyses led to the same patterns as in the main analyses, suggesting that the differences were mainly driven by the barriers and facilitating factors (Table S4 in Multimedia Appendix 5). Moreover, the skilled support intervention resulted in a negative effect on the self-reported MVPA in some sensitivity analyses.

### Exploratory Analyses

We conducted 3 exploratory analyses to gain insights into the effects of the type, timing, and temporal dynamics of the dyadic JITAIs (Tables 6-8). Regarding the type of dyadic JITAIs (ie, cross-over or joint), the results showed significant effects of cross-over JITAIs for both targeting the actor as well as the partner on device-based and self-reported MVPA. That means that on days when cross-over dyadic JITAIs were sent, couples were more physically active. Joint JITAIs did not increase device-based but increased self-reported MVPA, indicating that on days when joint dyadic JITAIs were sent, couples reported being more physically active. Furthermore, the effect sizes of the cross-over and joint JITAIs were comparable for both device-based and self-reported MVPA.

**Table 6:** Effects of the exploratory analyses of cross-over and joint just-in-time adaptive interventions on device-based and self-reported moderate-to-vigorous physical activity.

Parameter^a^	Device-based MVPA^b,c^				Self-reported MVPA^d^		
	Estimate, b (SE)	90% CI	*P* value^e^	Estimate, b (SE)		90% CI	*P* value^e^
Intercept	107.64 (3.75)	101.30 to 113.98	<.001	30.17 (3.49)		24.27 to 36.08	<.001
Cross-over JITAI_Actor_^f,g^	12.31 (4.16)	5.26 to 19.34	.006	13.68 (4.24)		6.51 to 20.86	.003
Cross-over JITAI_Partner_^h^	8.68 (4.24)	1.50 to 15.86	.049	11.91 (5.22)		3.09 to 20.74	.03
Joint JITAI^i^	8.87 (6.00)	−1.30 to 19.04	.15	19.20 (9.56)		3.03 to 35.69	.05
Time^j^	−1.59 (0.78)	−2.91 to −0.28	.049	−2.06 (0.78)		−3.37 to −0.75	.01
Wear time^k^	0.16 (0.01)	0.13 to 0.18	<.001	—^l^		—	—

^a^Number of couples=38; number of days=55.

^b^MVPA: moderate-to-vigorous physical activity.

^c^Number of cases in the analysis for device-based moderate-to-vigorous physical activity=3706.

^d^Number of cases in the analysis for self-reported moderate-to-vigorous physical activity=3944.

^e^All *P* values are 2-tailed.

^f^JITAI: just-in-time adaptive intervention.

^g^Days on which a cross-over JITAI targeted the actor’s MVPA (coded 1) were compared to days without cross-over JITAIs targeting the actors’s MVPA (coded 0).

^h^Days on which a cross-over JITAI targeted the partner’s MVPA (coded 1) were compared to days without cross-over JITAIs aiming at the partner’s MVPA (coded 0).

^i^Days on which a joint JITAI targeted both’s MVPA (coded 1) were compared to days without joint JITAIs (coded 0).

^j^Time was grand-mean-centered per 7 days.

^k^Wear time of the accelerometers in minutes was grand-mean-centered.

^l^Not applicable.

**Table 7:** Effects of the exploratory analyses of the timing of the dyadic just in-time adaptive interventions on device-based and self-reported moderate-to-vigorous physical activity.

Parameter^a,b,c^	Device-based MVPA^d,e^				Self-reported MVPA^f^		
	Estimate, b (SE)	90% CI	*P* value^g^	Estimate, b (SE)		90% CI	*P* value^g^
Intercept	107.65 (3.72)	101.34 to 113.97	<.001	30.16 (3.45)		24.30 to 36.02	<.001
Dyadic JITAI^h^ planning_Actor_	16.59 (4.74)	8.54 to 24.64	.002	17.92 (6.15)		7.48 to 28.36	.007
Dyadic JITAI activity_Actor_	−6.82 (5.37)	−15.94 to 2.30	.21	10.87 (6.39)		0.03 to 21.71	.10
Dyadic JITAI evening_Actor_	4.17 (3.29)	−1.42 to 9.77	.22	−1.69 (6.11)		−12.06 to 8.68	.74
Dyadic JITAI planning_Partner_	6.57 (5.11)	−2.11 to 15.25	.21	13.97 (6.18)		3.47 to 24.46	.03
Dyadic JITAI activity_Partner_	8.07 (5.65)	−1.53 to 17.67	.16	9.55 (2.99)		4.48 to 14.62	.003
Dyadic JITAI evening_Partner_	3.54 (3.82)	−2.95 to 10.03	.36	5.41 (3.43)		−0.41 to 11.24	.12
Time^i^	−1.66 (0.77)	−2.98 to −0.34	.04	−2.15 (0.69)		−3.33 to −0.98	.004
Wear time^j^	0.16 (0.01)	0.13 to 0.18	<.001	—^k^		—	—

^a^Number of couples=38; number of days=55.

^b^The “Actor” and “Partner” subscripts indicate who was targeted by the dyadic just-in-time adaptive intervention. For just-in-time adaptive interventions targeting the actor, days on which a dyadic just-in-time adaptive intervention targeted the actor’s moderate-to-vigorous physical activity (coded as 1) were compared with days without dyadic just-in-time adaptive interventions targeting the actor’s moderate-to-vigorous physical activity (coded as 0). For just-in-time adaptive interventions targeting the partner, days on which a dyadic just-in-time adaptive intervention targeted the partner’s moderate-to-vigorous physical activity (coded as 1) were compared with days without dyadic just-in-time adaptive interventions targeting the partner’s moderate-to-vigorous physical activity (reference category, coded as 0).

^c^Dyadic just-in-time adaptive interventions may be triggered before the planning intervention (labeled planning), before a planned activity (labeled activity), and in the evening (labeled evening).

^d^MVPA: moderate-to-vigorous physical activity.

^e^Number of cases in the analysis for device-based moderate-to-vigorous physical activity=3706.

^f^Number of cases in the analysis for self-reported moderate-to-vigorous physical activity=3944.

^g^All *P* values are 2-tailed.

^h^JITAI: just-in-time adaptive intervention.

^i^Time was grand-mean-centered per 7 days.

^j^Wear time of the accelerometers in minutes was grand mean-centered.

^k^Not applicable.

**Table 8:** Effects of the exploratory analyses of the lagged effects of the dyadic just-in-time adaptive interventions on device-based and self-reported moderate-to-vigorous physical activity.

Parameter^a,b,c^	Device-based MVPA^d,e^			Self-reported MVPA^f^		
	Estimate, b (SE)	90% CI	*P* value^g^	Estimate, b (SE)	90% CI	*P* value^g^
Intercept	107.63 (3.73)	101.28 to 113.97	<.001	30.05 (3.51)	24.08 to 36.02	<.001
Dyadic JITAI_Actor_^h^	7.79 (3.12)	2.47 to 13.10	.02	9.23 (3.44)	3.38 to 15.07	.01
Dyadic JITAI_Actor lag1_	5.69 (3.28)	0.10 to 11.27	.09	10.71 (3.41)	4.90 to 16.51	.004
Dyadic JITAI_Actor lag2_	0.22 (2.55)	−4.13 to 4.56	.93	6.38 (2.65)	1.87 to 10.89	.02
Dyadic JITAI_Actor lag3_	1.13 (4.27)	−6.14 to 8.40	.79	5.25 (5.16)	−3.52 to 14.03	.31
Dyadic JITAI_Partner_	4.10 (4.04)	−2.79 to 10.99	.32	7.66 (4.27)	0.39 to 14.92	.08
Dyadic JITAI_Partner lag1_	3.22 (2.87)	−1.68 to 8.11	.27	4.35 (2.68)	−0.20 to 8.91	.12
Dyadic JITAI_Partner lag2_	1.61 (3.18)	−3.80 to 7.03	.62	−0.96 (2.76)	−5.65 to 3.73	.73
Dyadic JITAI_Partner lag3_	0.44 (4.10)	−6.54 to 7.42	.92	−2.33 (4.35)	−9.73 to 5.07	.60
Time^i^	−1.82 (0.83)	−3.23 to −0.41	.04	−2.56 (0.73)	−3.81 to −1.31	.002
Wear time^j^	0.16 (0.01)	0.13 to 0.18	<.001	—^k^	—	—

^a^Number of couples=38; number of days=55.

^b^The “Actor” and “Partner” subscripts indicate who was targeted by the dyadic just-in-time adaptive intervention. For just-in-time adaptive interventions targeting the actor, days on which a dyadic just-in-time adaptive intervention targeted the actor’s moderate-to-vigorous physical activity (coded as 1) were compared with days without dyadic just-in-time adaptive interventions targeting the actor’s moderate-to-vigorous physical activity (coded as 0). For just-in-time adaptive interventions targeting the partner, days on which a dyadic just-in-time adaptive intervention targeted the partner’s moderate-to-vigorous physical activity (coded as 1) were compared with days without dyadic just-in-time adaptive interventions targeting the partner’s moderate-to-vigorous physical activity (coded as 0).

^c^The 3 lag parameters indicate how many occasions ago the just-in-time adaptive interventions targeted the moderate-to-vigorous physical activity.

^d^MVPA: moderate-to-vigorous physical activity.

^e^Number of cases in the analysis for device-based moderate-to-vigorous physical activity=3706.

^f^Number of cases in the analysis for self-reported moderate-to-vigorous physical activity=3944.

^g^All *P* values are 2-tailed.

^h^JITAI: just-in-time adaptive intervention.

^i^Time was grand mean-centered per 7 days.

^j^Wear time of the accelerometers in minutes was grand mean-centered.

^k^Not applicable.

The dyadic JITAIs varied in effectiveness depending on the time they were sent. The dyadic JITAIs sent before the planning intervention targeting the actor increased the device-based and self-reported MVPA. The dyadic JITAIs sent before the planning intervention targeting the partner increased the self-reported but not the device-based MVPA. Furthermore, the dyadic JITAIs sent before the planned activity targeting the actor did not increase device-based nor self-reported MVPA. The dyadic JITAIs sent before the planned activity targeting the partner increased self-reported, but not the device-based MVPA. Finally, neither the dyadic JITAIs sent in the evening targeting the actor nor the partner had any significant effect on the self-reported or device-based MVPA.

Regarding the temporal dynamics of the JITAIs, there was evidence for lagged effects of the dyadic JITAIs targeting the actor on self-reported and, to a lesser extent, on device-based MVPA. This finding indicates that the dyadic JITAIs targeting the actor not only affected the MVPA at the time they were initially aimed at, but their effects lasted and influenced subsequent self-reported MVPA. There was no evidence of lagged effects of dyadic JITAIs targeting the partner on device-based or self-reported MVPA.

## Discussion

### Principal Findings

This study demonstrated the promising effects of smartphone-based dyadic interventions in promoting MVPA in romantic partners. Participants reported higher device-based and self-reported MVPA during the intervention phase than during the control phase. Furthermore, planning physical activities increased the participants’ self-reported but not device-based MVPA. Dyadic JITAIs targeting the actor as well as the partner increased the device-based and self-reported MVPA. However, sensitivity analyses indicated that some of these effects were not robust, suggesting that contextual factors play a relevant role in the effectiveness of dyadic JITAIs. Exploratory analyses showed that cross-over JITAIs promoted device-based and self-reported MVPA, and joint JITAIs promoted self-reported MVPA. Moreover, dyadic JITAIs aiming at different times (ie, before a planning intervention, before a planned activity, and in the evening) were differently effective in increasing MVPA. Finally, there was evidence for lagged effects of the dyadic JITAIs targeting the actor for device-based and self-reported MVPA but not for lagged effects of dyadic JITAIs targeting the partner.

### Effects of the Dyadic Interventions

Consistent with past research, this study found promising effects of dyadic interventions to increase physical activity. Overall, the complex intervention, including different dyadic interventions, was effective in increasing both device-based and self-reported MVPA (hypothesis 1). However, there was heterogeneity in the effects of the intervention phase across couples. This heterogeneity may reflect differences in couple dynamics or insufficient knowledge of moderating factors interacting with the intervention or control phase [48]. A better understanding of these factors may help improve the effectiveness of dyadic interventions [39,56]. Future research may investigate intrapersonal characteristics, relationship dynamics, and the broader social context to provide valuable insights into factors relevant to intervention development and implementation [56].

This study found significant effects of the planning intervention on the self-reported but not device-based MVPA (hypothesis 2). These results are comparable with previous studies that found mixed effects of dyadic and collaborative planning [28,31,32,35]. In contrast to previous studies, this study used a within-couple design and compared days with planned activities to days without planned activities instead of comparing the effects of various planning interventions (eg, dyadic, collaborative, and individual) on subsequent physical activity. Additionally, the participants could freely choose and vary if they wanted to plan dyadically or collaboratively. This approach aimed to enable participants to plan according to their needs and preferences to compensate for potential challenges associated with each form of planning. However, these attempts to better tailor the planning interventions to couples’ needs and to use more contingent outcomes [57] were not sufficient to enhance the planning intervention’s effectiveness, at least with regard to the device-based measure of MVPA. As discussed previously [58], it seems necessary to identify under what circumstances and for whom these forms of planning unfold the most beneficial effects.

To our knowledge, our study was the first to implement dyadic JITAIs to promote MVPA in romantic couples. In line with previous studies on the effectiveness of JITAIs on health behaviors [39], this study showed promising effects of dyadic JITAIs in promoting MVPA (hypothesis 3a). Furthermore, not only the dyadic JITAIs targeting the actor but also those targeting the partner increased the MVPA (hypothesis 3b). This finding may be explained by both the dyadic nature of the JITAIs as well as the interdependence between close partners in a romantic relationship [6]. One partner’s engagement may motivate the other partner to engage in physical activity as well [59]. Furthermore, helping the partner engage in physical activity may have positive outcomes for the provider [43]. For example, providing social support or control to the partner may reiterate the importance of physical activity for the provider [60], increase self-esteem [61], and contribute to feeling more energized [62], promoting engagement in physical activity. Thus, deciding who to target with dyadic interventions appears to be an essential design choice when developing and implementing dyadic interventions.

The effect of the planning and the effect of the dyadic JITAIs targeting the actor on self-reported MVPA disappeared when controlling for additional covariates. Exploratory analyses suggested that these discrepancies were driven by the reported barriers and facilitating factors. Potentially, the barriers and facilitating factors may act as mediators in explaining the JITAIs’ effects on MVPA. The planning and JITAIs prompted processes to help engage in MVPA, which may have removed barriers or facilitated engagement in MVPA. Furthermore, it is also possible that the causality goes in the other direction, in that engagement and nonengagement in MVPA influenced the perception of barriers and facilitating factors.

We found an unexpected negative effect of the skilled support intervention on self-reported MVPA in some of the analyses. However, it is important to note that this part of the intervention was not randomized.

This study found some differences in the effects of the dyadic interventions on device-based and self-reported MVPA. Interestingly, in this study, the device-based measure of MVPA was much higher than the self-reported measure. These 2 measures represent related but distinct indicators of MVPA [63]. There are several explanations for these differences. First, self-reported and device-based MVPA may capture different aspects of physical activity [64]. For example, self-reported physical activity may refer more closely to the activities perceived as physical activity by the participants, whereas device-based physical activity may also include other forms of movement, such as naturally-occurring MVPA. Furthermore, it may be possible that engaging in planned physical activities (captured by both the device-based and self-reported MVPA) leads to compensatory effects in that the engagement in naturally occurring MVPA (captured only by device-based MVPA) decreases [65], leading to some discrepancies between the measures. Second, there may be response biases in the self-reported but not device-based MVPA measure [64]. Participants may indicate higher MVPA if they received an intervention because they expect it to be effective. Finally, the device-based MVPA may have misclassified some motions as MVPA, which would also explain why the device-based MVPA was substantially higher than the self-reported MVPA [65].

### Exploratory Analyses

Results from the exploratory analyses showed that cross-over JITAIs increased device-based and self-reported MVPA, and joint JITAIs increased self-reported MVPA while having similar effect sizes. Given the exploratory nature of our analyses, results need to be replicated in confirmatory designs and analyses in the future. In addition to examining the main effects of these different prototypes of dyadic interventions [10] and despite their comparable effect sizes found in our study, these 2 types of interventions may target different mechanisms of action [10,14]. For example, joint interventions, such as discussions and collaborative planning, may trigger interactions in which both partners are equally involved. In contrast, cross-over interventions, such as prompting to provide social support and to exert social control, may trigger one-sided interactions where one partner is more involved than the other. Future research is needed to better understand the potential different effects of cross-over and joint interventions and how they may vary under specific circumstances.

The second set of exploratory analyses showed that dyadic JITAIs with different timings varied in effectiveness. Specifically, there was stronger evidence for the effectiveness of the dyadic JITAIs sent before the planning intervention and before the planned activity than those sent in the evening. These dyadic JITAIs targeted different aspects of the behavior change process. The dyadic JITAIs before the planning interventions aimed at increasing the quality of the dyadic and collaborative plans, a factor central to the effectiveness of planning [58]. The dyadic JITAIs before the planned activity aimed at helping to engage in this activity. Sending dyadic JITAIs at this specific time may be effective because they directly enhance commitment to the planned activities. The dyadic JITAIs in the evening aimed at the partners to reflect on their goal progress, discussing things that went well or poorly regarding physical activity, or prompting support or positive control in the future. A reason for the lack of effectiveness may be that these dyadic JITAIs were not matched to a particular state of vulnerability or opportunity since they did not target immediate preparation or engagement in MVPA directly. Furthermore, the participants may not have had the opportunity to engage in the prompted task because the interventions were sent too late in the day. Thus, future studies should also examine intervention fidelity and intervention engagement, which may vary with different timings of the interventions.

The final exploratory analyses showed that dyadic JITAIs may have effects over and above their targeted time point. Implementing dyadic JITAIs to improve the social exchange processes may translate to improved interaction patterns that sustainably facilitate engagement in health behaviors [11]. Since many health behaviors, such as physical activity, require consistent engagement over extended periods of time, this would be promising for promoting longer-term behavior change [66]. Specifically, this study found evidence for long-term effects of dyadic JITAIs targeting the actor but not for those targeting the partner. These results suggest that different mechanisms may explain these effects [43]. For example, receiving social support and control may establish a subjective norm or change attitudes regarding physical activity [67], which translates into regular engagement in physical activity. In contrast, providing social support and control may promote MVPA by increasing positive effects [43] and energy levels [61], which may entail more transient effects. Future research is needed to investigate these underlying mechanisms in more detail.

Past research has illustrated a need to better understand the boundary conditions moderating the effectiveness of (dyadic) JITAIs [39]. The exploratory analyses of this study contribute to this call by illustrating preliminary insights into the importance of the type and timing of the dyadic JITAIs for their effectiveness. Additionally, it showed the potential long-term effects of dyadic JITAIs, providing new insights and opportunities for health behavior promotion. Given the exploratory nature, however, the next steps are to replicate findings with confirmatory analyses. Moreover, in future studies with more participants, it might also be worthwhile to examine interactions between the different factors examined here. For example, it might well be that joint JITAIs work better when being sent in the evening to prompt joint reflection of past interactions, compared with cross-over JITAIs that might work better before a planning intervention. It is also possible that joint JITAIs might have longer-lasting effects, as the positive dyadic dynamics in supportive interactions promoted by these kinds of dyadic JITAIs might add to the maintenance of joint behavior change attempts.

### Strengths and Limitations

This study has various strengths. First, the study investigated health behavior change from a dyadic rather than individual perspective, allowing for a more complete understanding of health behavior change processes in romantic relationships. Second, the microrandomized trial design allowed us to experimentally investigate the dyadic health behavior change as a process over time and to examine daily dynamics [47]. This also allowed us to examine the causal effects of the interventions on the outcomes [50]. Third, we were able to investigate shorter-term as well as lagged effects of the dyadic JITAIs. Finally, including device-based and self-reported MVPA measurements offered a more comprehensive understanding of the effects of dyadic interventions on MVPA [63], as well as addressed potential methodological challenges inherent to the measurement methods.

Despite these strengths, this study also has its limitations. As it was a pilot study, the sample size of 38 romantic couples was relatively small. This relatively small sample size may limit the statistical power, which may be especially the case for the exploratory analyses, where there have been fewer instances of interventions. However, the relatively large number of days examined per couple (ie, 55 days) partly compensates for this small sample size [47]. The limited number of couples also restricted the possibility of conducting more detailed analyses exploring the effectiveness of interventions targeting specific social exchange processes (eg, emotional support and positive social control) and potential mechanisms underlying the intervention effects or interaction effects as outlined above. Furthermore, there were different types of dyadic interventions (ie, skilled support intervention, planning interventions, and JITAIs) implemented in the study. These interventions were designed to build upon and facilitate each other. For example, the dyadic JITAIs were built on the knowledge of skilled support and were triggered in reference to the planned activities. However, this also implies that it remains unclear how exactly and to what extent these different dyadic interventions influenced each other (eg, if the dyadic JITAIs would have been effective without an initial skilled support intervention). Finally, the study targeted inactive romantic couples who expressed a willingness to increase their physical activity. Thus, the study sample was self-selected and therefore may not fully represent the general internet population.

### Conclusion

This study extended the common approach of promoting health behavior change of individuals by including partners in the behavior change process. This allowed us to implement DBCTs that address social interactions between the partners. Overall, this study demonstrated the effectiveness of dyadic interventions, including planning interventions and dyadic JITAIs. Furthermore, the exploratory analyses provided first hints to the assumptions that conditions, such as the type, timing, and target, moderate the effectiveness of dyadic JITAIs, providing new insights into conceptual and design elements of dyadic JITAIs. Future research is needed to get a more comprehensive understanding of the effectiveness of dyadic interventions. Specifically, the moderating variables influencing the effectiveness of dyadic interventions and the mechanisms explaining dyadic interventions should be examined.

## Appendix

Multimedia Appendix 1 CONSORT-eHEALTH checklist (V 1.6.1).

Multimedia Appendix 2 Informed Consent.

Multimedia Appendix 3 Screenshots Study App.

Multimedia Appendix 4 Dyadic Just-in-time Adaptive Interventions.

Multimedia Appendix 5 Additional Results.

## Abbreviations

CONSORT: Consolidated Standards of Reporting Trials
CONSORT-EHEALTH: Consolidated Standards of Reporting Trials of Electronic and Mobile Health Applications and Online Telehealth
DBCT: dyadic behavior change technique
JITAI: just-in-time adaptive intervention
MVPA: moderate-to-vigorous physical activity
OSF: Open Science Framework

## Data Availability

Data are available upon reasonable request.
