# Supplementary material for: Effectiveness of Smartphone-Based Dyadic Interventions to Increase Physical Activity in Romantic Couples: Microrandomized Trial
Source: JMIR Mhealth Uhealth. 2026 Jan 27;14:e67136. doi: 10.2196/67136 (PMC12892032; doi:10.2196/67136)
Supplement: Multimedia Appendix 2 [file mhealth_v14i1e67136_app2.docx]

**Einwilligungserklärung**

Bitte lesen Sie das folgende Formular sorgfältig durch und kontaktieren Sie das Studienteam bei Fragen.

**Schriftliche Einwilligungserklärung zur Teilnahme an der Studie „Gemeinsam Aktiv! – Mehr Bewegung in der Partnerschaft“**

- Ich wurde von der Studienleitung schriftlich über den Zweck, Ablauf der Studie, über mögliche Vorteile sowie über eventuelle Risiken informiert.
- Ich habe die schriftlichen Studieninformationen zur oben genannten Studie gelesen und verstanden.
- Falls bei Ihnen oder Ihrer Partnerin / Ihrem Partner gesundheitliche Gründe bekannt sind, welche dagegen sprechen, sich körperlich zu betätigen, oder falls Sie diesbezüglich unsicher sind, haben Sie mit Ihrem Hausarzt oder Ihrer Hausärztin abgeklärt, ob und in welchem Rahmen Sie Sport treiben können. Die Teilnahme geschieht auf eigene Verantwortung.
- Ich weiss, dass durch den Upload von Daten im Hintergrund über die Apps, je nach Vertrag mit einem Netzanbieter, Kosten entstehen können. Mir werden diese zusätzlichen Kosten von der Studienleitung gegen entsprechenden Nachweis erstattet.
- Ich nehme an dieser Studie freiwillig teil. Ich kann jederzeit und ohne Angabe von Gründen meine Zustimmung zur Teilnahme widerrufen, ohne dass mir deswegen Nachteile entstehen.
- Ich bin über die Datenschutzmassnahmen der oben genannten Studie informiert worden und stimme zu, dass meine Daten für die beschriebenen Zwecke verwendet und gespeichert werden dürfen.
- Ich weiss, dass ich jederzeit (bis zum Löschen aller Kontaktdaten) ohne Angabe von Gründen das Löschen aller meiner Daten verlangen kann, ohne dass mir dabei Nachteile entstehen.
- Ich hatte genügend Zeit, meine Entscheidung zu treffen.

Ich stimme zu

Bitte geben Sie ebenfalls Ihre Zustimmung, dem Betreiber dieser Software das Verwenden und Speichern Ihrer Daten, im Rahmen dieses Projektes, zu gestatten.

Ich akzeptiere die [Datenschutzrichtlinie](https://brightanswer.ch/privacy)
