# Supplementary figures and images for: Effectiveness of Smartphone-Based Dyadic Interventions to Increase Physical Activity in Romantic Couples: Microrandomized Trial
Source: JMIR Mhealth Uhealth. 2026 Jan 27;14:e67136. doi: 10.2196/67136 (PMC12892032; doi:10.2196/67136)

## Slide 1
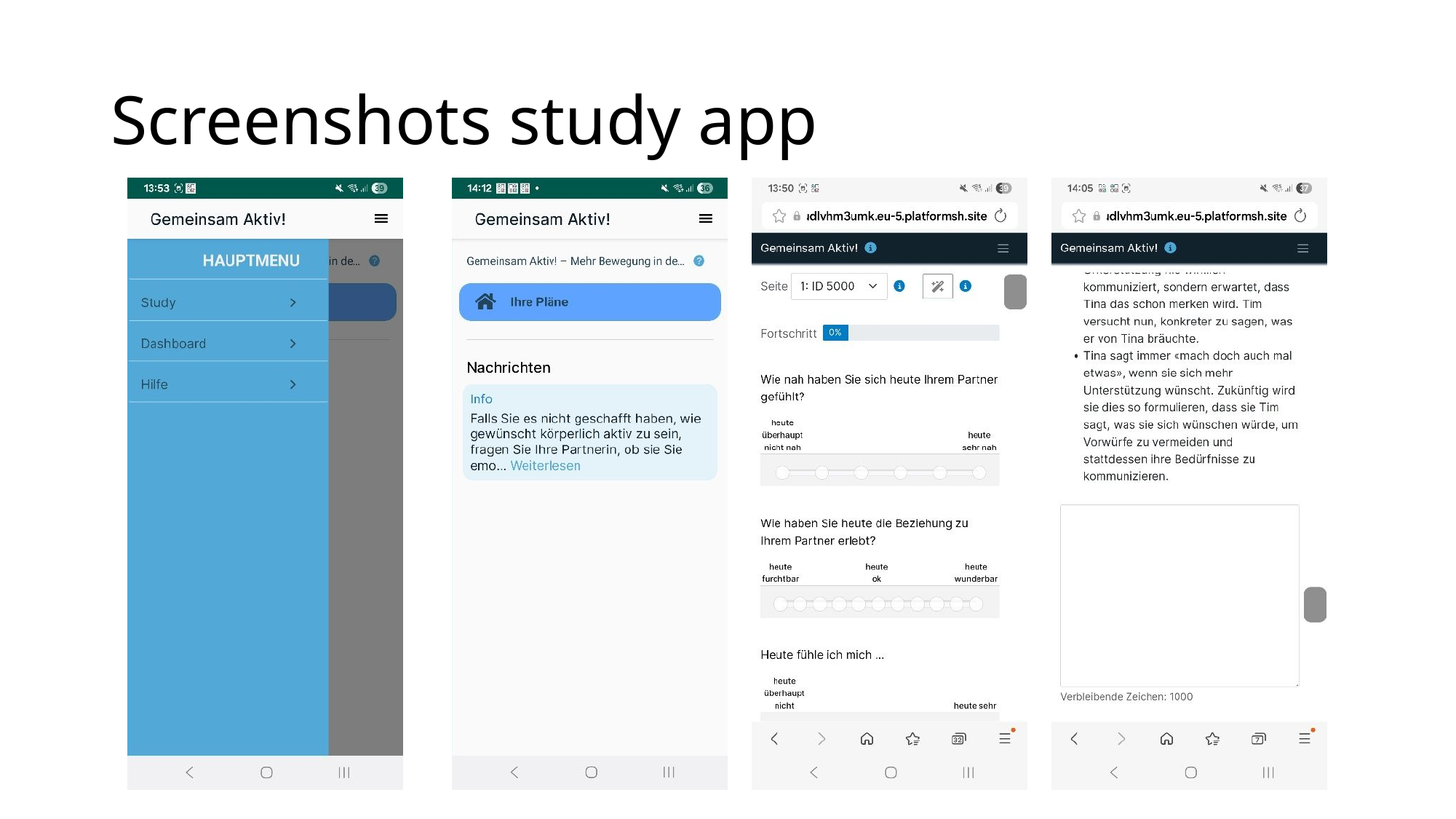

# Screenshots study app

Supplement: Multimedia Appendix 3 [file mhealth_v14i1e67136_app3.pptx]
