# Supplementary material for: Effectiveness of Smartphone-Based Dyadic Interventions to Increase Physical Activity in Romantic Couples: Microrandomized Trial
Source: JMIR Mhealth Uhealth. 2026 Jan 27;14:e67136. doi: 10.2196/67136 (PMC12892032; doi:10.2196/67136)
Supplement: Multimedia Appendix 4 [file mhealth_v14i1e67136_app4.docx]

This document outlines a comprehensive description of the implementation of the dyadic just-in-time adaptive interventions (JITAIs) in the pilot study of the Time and Ties project. The documentation of the entire Time and Ties project can be found on OSF. The dyadic JITAIs were delivered through the participants’ smartphones and targeted the social exchange processes within romantic relationships that were hypothesised to facilitate engagement in moderate-to-vigorous physical activity (MVPA). The dyadic JITAIs were tailored based on the situations and problems the couples were facing. Depending on the content of the intervention, the dyadic JITAIs were sent to either or both partners and targeted either or both partners.

## **Overview of the Intervention Components**

Figure S1 illustrates the various intervention components and measurements used in the study. The device-based and self-reported MVPA were measured throughout the whole eight-week study period. Furthermore, all groups received a skilled support intervention at the beginning of their intervention phase. Group A received the dyadic interventions (i.e., planning intervention and dyadic JITAIs) throughout the remaining seven weeks; group B received the dyadic interventions in the last three weeks of the study; and group C received the dyadic interventions in the first three weeks after the baseline phase.

**Figure S1.** Overview of the Study Components.


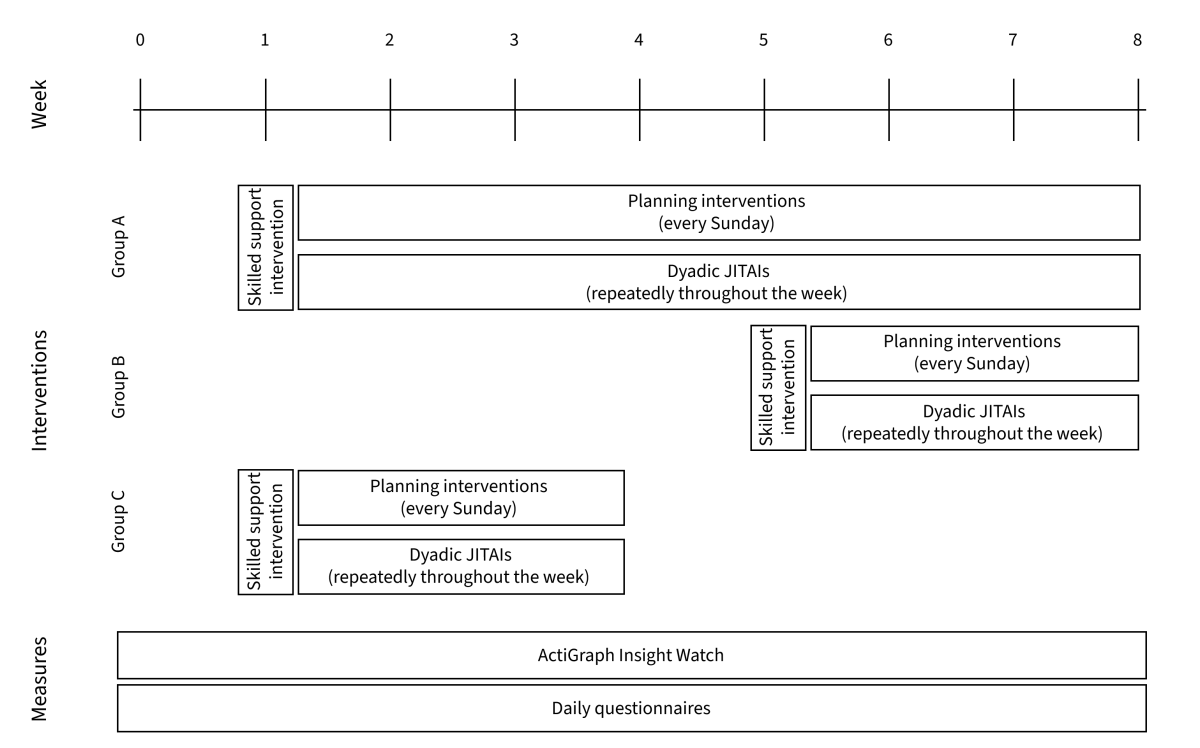


## **Components of the Dyadic JITAIs**

In this section, we outline the key components of the dyadic JITAIs, following the guidelines proposed by Nahum-Shani et al. [1] and Qian et al. [2] for reporting the design elements of the JITAIs.

### ***State of Vulnerability and Opportunity***

The state of vulnerability and the state of opportunity describe periods of heightened susceptibility to adverse health outcomes (vulnerability) or positive health outcomes (opportunity), respectively [1]. In the Time and Ties pilot study, we identified the following three states of opportunities to send dyadic JITAIs:

- A dyadic JITAI may be sent before the planning intervention to make the joint goal-setting and planning of physical activities more adequate and ambitious.
- A dyadic JITAI may be sent before a planned activity to positively influence the decision to engage or not engage in the planned physical activity.
- A dyadic JITAI may be sent in the evening to trigger reflections about the (lack of) MVPA and goal progress made during the day.

### ***Proximal Outcomes***

The proximal outcome describes the short-term goals of the interventions [1]. These proximal outcomes can be mediators or intermediate measures of the distal outcome. In the Time and Ties pilot study, the proximal outcome was to increase both partners’ daily levels of MVPA.

### ***Distal Outcome***

The distal outcome describes the long-term, ultimate goal the interventions try to achieve [1]. In the Time and Ties pilot study, the distal outcome was to increase both partners’ health.

### ***Intervention Options***

The intervention options describe all possible treatments employed at a decision point [1]. In the Time and Ties pilot study, various text messages were sent to the participants’ smartphones with the aim of improving the social exchange processes between the partners hypothesised to promote MVPA. The intervention options were either to send one of various text messages or not to send any text message. Table S3 contains some examples of the intervention options (a complete list can be found on OSF.

### ***Decision Points***

The decision points describe the time when a decision is made to send or not send an intervention [1]. In the Time and Ties pilot study, every evening after completing the daily questionnaire, a dyadic JITAI per couple was selected based on the responses in the daily questionnaires of both partners. The stored intervention was subsequently triggered at the corresponding predefined time during a state of opportunity or vulnerability (i.e., before the planning intervention, before the planned activity, and in the evening).

### ***Tailoring Variables***

The tailoring variables describe the information used to tailor interventions (e.g., deciding when and how to intervene) [1]. In the Time and Ties pilot study, we chose variables that informed about social exchange processes, psychological states, and resources that were hypothesised to be relevant to engaging in MVPA. All tailoring variables were assessed in the daily and weekly questionnaires (see Table S1, please note that all item examples are roughly translated from German).

Table S1. List of all tailoring variables used to inform the JITAIs.

| Variable |  | Item | |
| --- | --- | --- | --- |
|  | Questionnaire | Question^a^ | Response option^b^ |
|  |  |  |  |
| Planning satisfaction | Weekly | Overall, how satisfied are you overall with the planning process? | 0 = very dissatisfied  5 = very satisfied |
| Moderate-to-vigorous physical activity | Daily | Today I was physically active, ...  (please select all that apply) | 0 = ... exactly as planned.  1 = ... longer than planned.  2 = ... but shorter than planned.  3 = ... but with a different activity than planned.  4 = ... with an unplanned activity (spontaneous). |
|  |  | Today I was not physically active, ...  (please select all that apply) | 0 = ... because no activity was planned for today.  1 = ... although an activity was planned for today. |
| Instrumental evaluation | Weekly | How would you rate the advantages and disadvantages of being physically active for you personally?  I think that... | ­−5 = disadvantages outweigh by far.  0 = advantages and disadvantages balance each other out.  5 = advantages outweigh by far. |
| Affective outcome expectancy | Daily | How do you feel today when you think about your next physical activity? | −5 = very bad today  0 = neither  5 = very good today |
| Self-efficacy | Daily | I am confident that I can manage to be physically active tomorrow, even if it will be difficult. | 0 = not true at all today  5 = completely true today |
| Available resources | Daily | Being physically active tomorrow would/will be ... | 0 = ... impossible (important prerequisites are not fulfilled)  3 = ... possible with some effort (several prerequisites are not fulfilled)  6 = ... possible without any problems (all prerequisites are fulfilled) |
| Commitment | Daily | I am determined to do everything I can tomorrow to achieve my goal of being physically active, even if it takes great effort. | 0 = not true at all today  5 = completely true today |
| Positive social control | Daily | Through persuasion (e.g., well-intentioned suggestions, constructive discussions, imparting information, etc.), my partner today tried to get me to be more physically active. | 0 = not true at all today  5 = completely true today |
| Sabotage | Daily | Through persuasion (e.g., well-intentioned suggestions, constructive discussions, imparting information, etc.), my partner tried to get me to be less physically active today. | 0 = not true at all today  5 = completely true today |
|  | Daily | By exerting pressure (e.g., nagging, reproaching, insinuating, making me feel guilty, etc.), my partner tried to get me to be less physically active today. | 0 = not true at all today  5 = completely true today |
| Timing of positive social control /sabotage | Daily | Today, when did your partner try to convince you to be more / less physically active? (please select all that apply) | 1 = during the planning of the physical activity  2 = just before or while you were planning to be physically active  3 = at another time |
| Negative social control | Daily | By exerting pressure (e.g., nagging, reproaching, insinuating, making me feel guilty, etc.), my partner tried to get me to be more physically active today. | 0 = not true at all today  5 = completely true today |
| Timing of negative social control /sabotage | Daily | When did your partner pressure you to be more or less physically active? (please select all that apply) | 1 = during the planning of the physical activity  2 = just before or while you were planning to be physically active  3 = at another time |
| Emotional support (comfort) | Daily | How much did your partner cheer you up today when you were less physically active than you would have liked? | 0 = not at all today  5 = very much today |
| Emotional support (reassurance) | Daily | How much did your partner reassure you today that it isn’t so bad if you are less physically active than you had planned together? | 0 = not at all today  5 = very much today |
| Timing of emotional support (comfort /reassurance) | Daily | When did your partner cheer you up about your physical activity today? (please select all that apply) | 0 = during the planning of the physical activity  1 = today during the day before you were/were going to be physically active  2 = just before you wanted to be physically active  3 = while you were/were going to be physically active  4 = shortly after you were/wanted to be physically active  5 = later, after you were/wanted to be physically active |
| Emotional support (joy) | Daily | How much has your partner supported you emotionally today, making it more fun for you to be physically active? | 0 = not at all today  5 = today very much |
| Timing of emotional support (joy) | Daily | When did your partner support you emotionally today, making it more fun for you to be physically active? (please select all that apply) | 0 = during the planning of the physical activity  1 = today during the day before you were/were going to be physically active  2 = just before you wanted to be physically active  3 = while you were/were going to be physically active  4 = shortly after you were/wanted to be physically active  5 = later, after you were/wanted to be physically active |
| Practical support | Daily | Practical support (e.g., guidance or help in doing the activity, driving to the gym, taking over chores to make more time for physical activity, etc.) can make it easier or even possible to be physically active.  How much did your partner practically support you today? | 0 = not at all today  5 = today very much |
| Convincing about self-efficacy | Weekly | In the past week, how hard has your partner tried to convince you that you can or cannot be physically active? | −5 = very much that I can not do it  0 = not at all  5 = very much that I can make it |
| Timing of convincing about self-efficacy | Weekly | Last week, when did your partner try to convince you that you can manage to be more or less physically active? (please select all that apply) | 1 = during the planning of the physical activity  2 = just before or while you were planning to be physically active  3 = at another time |
| Convincing about advantages | Weekly | How much has your partner tried to convince you of the advantages or disadvantages of physical activity during the past week? | -5 = tried very hard to convince me of less advantages and more disadvantages  0 = not tried to convince me  5 = very strongly tried to me convince of more advantages and less disadvantages |
| Emphasising commitment | Daily | Today, how much did your partner push you to be physically active according to the plan you made together? | 0 = not at all today  5 = today very much |

^a^The wording of the questionnaire items is a rough translation from German.

^b^The wording of the response options is a rough translation from German.

### ***Decision Rules***

The decision rules describe the operationalisation of how the intervention is triggered, for whom, and when [1]. In the Time and Ties pilot study, we developed an algorithm that chose the appropriate interventions. First, scores for various situations were calculated based on the tailoring variables. These situations describe problems that the couples were currently facing that prevent them from engaging in MVPA. Additionally, there were some situations in which the couple successfully engaged in their planned physical activity. Second, the algorithm chose an appropriate situation based on these scores, the recency (i.e., when was the situation chosen the last time) and the frequency (i.e., how often was the situation chosen so far) of the situation. Each situation is linked to a pool of different intervention options addressing the same problem (or success). In the last step, the algorithm chose an intervention option from this pool based on the recency (i.e., when was this intervention option chosen the last time) and the frequency (i.e., how often was the intervention option chosen so far) of the intervention option. A detailed description of the algorithm and a list of all situations and intervention options can be found on OSF.

### **Randomisation**

In traditional micro-randomised trials, there is a set probability at every decision point based on which an intervention is triggered [2]. However, in the Time and Ties pilot study, randomisation was followed on two levels. First, couples were randomly assigned to one of the three intervention groups (ensuring that the number of couples in each group was approximately equal). Each group had a different intervention phase when the couples received dyadic JITAIs. The first group received a seven-week intervention phase after an initial control phase. The second group received a three-week intervention phase after an initial control phase, followed by a four-week control phase without interventions. The third group received a three-week intervention phase at the end of the study only, thus starting with a four-week control phase without interventions.

Second, the dyadic JITAIs within the intervention phase were randomised. Dyadic JITAIs were selected from a pool of intervention options depending on how well they matched the couple’s situation and how frequently and recently a situation was selected. This pool also contained control interventions in which cases no intervention was sent. Thus, this algorithm varied interventions and non-interventions that allowed for testing the effectiveness of the interventions.

## **Observations of Context**

The observation of context includes variables of practical or scientific interest recorded at a decision point [2]. Various observations of context variables were included in the sensitivity analyses (Table S2).

Table S2. List of all observations of context variables.

| Variable | Description | Response option |
| --- | --- | --- |
|  |  |  |
| Study day | Day of the study | 0–54 |
| Weekend | Indicating if it is weekend or weekday | 0 = weekday 1 = weekend |
| Skilled support intervention | Indicating if the skilled support intervention was already completed or not. | 0 = before skilled support intervention 1 = after skilled support intervention |
| Planned activity | Indicating if there is a planned activity on the day. | 0 = no planned activity 1 = planned activity |
| Intervention group | Indicating the intervention group. | A = group A B = group B  C = group C |
| Wear time of accelerometer | Wear time of the CentrePoint InsightWatch accelerometer. | Wear time in minutes |
| Barriers | Score of all barriers of the day. | 0–40 |
| Facilitating factors | Score of all facilitating factors of the day. | 0–40 |

## **Targeted Days**

The days targeted by the dyadic JITAIs did not necessarily correspond to the days on which the dyadic JITAIs were sent. As described above, there were three different decision points for the dyadic JITAIs: 1) before the planning intervention, 2) in the morning of a planned activity, and 3) in the evening after completing the daily questionnaire. Depending on the decision point, the intervention targeted the MVPA on a different day. We use the notation proposed by Boruvka et al. [3] to illustrate which days were targeted by the interventions at each decision point:

- *X_t_* represents the time period when the context variables are gathered. This also includes the tailoring variables.
- *A_t_* represents the decision point of the dyadic JITAIs. At this point, it is decided which intervention option is sent.
- *Y_t+1_* represents the proximal outcome. In the case of the Time and Ties pilot study, this is the MVPA over the day.

The dyadic JITAIs before the planning intervention were based on the tailoring variables of the previous week. These intervention options aimed to improve the planning process during the planning intervention (i.e., goal setting, action planning, and coping planning) [4]. We expected these intervention options to improve the planning and, thus, make it more likely for couples to complete these plans and engage in the corresponding planned activities. Since the plans were made for one week, we expect the intervention options before the planning intervention to increase the MVPA on every day for which a physical activity was planned for the upcoming week (see Figure S2). Note that the self-reported MVPA was measured upon the point the participants completed the daily questionnaire (although we instructed the participants to complete the daily questionnaire when they no longer plan to be physically active), and the device-based MVPA was measured throughout the day until the end of the day. The Figure shows an example of a dyadic JITAI before the planning intervention. Here, the intervention option was triggered in the morning of the day with the planning intervention (A_t_) based on the information from the daily and weekly questionnaires from the past week (X_t_). The proximal outcomes are the levels of MVPA in the upcoming week on days when activities were planned (Y_t+1_). Note that the level of MVPA on days without planned activity is no proximal outcome.

**Figure S2.** Implementation of the dyadic JITIAs before the planning intervention.

**
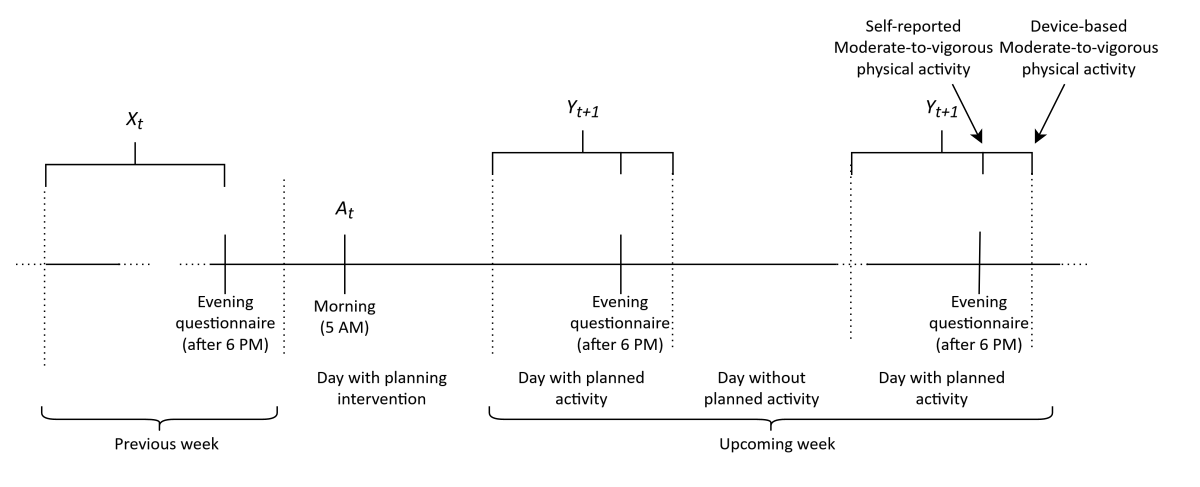
**

The dyadic JITAIs in the morning on days when a physical activity was planned were based on the responses from the daily questionnaire from the previous evening. These intervention options aimed to increase engagement in the planned physical activity. Thus, they targeted the MVPA on the same day as they were sent (see Figure S3). The Figure shows an example of a dyadic JITAI before the planned activity. Here the JITAI was triggered in the morning of the day with a planned activity (A_t_) based on the information from the daily questionnaire from the past day (X_t_). The proximal outcomes are the levels of MVPA on the same (Y_t+1_).

**Figure S3.** Implementation of the dyadic JITIAs before the planned physical activity.^a^


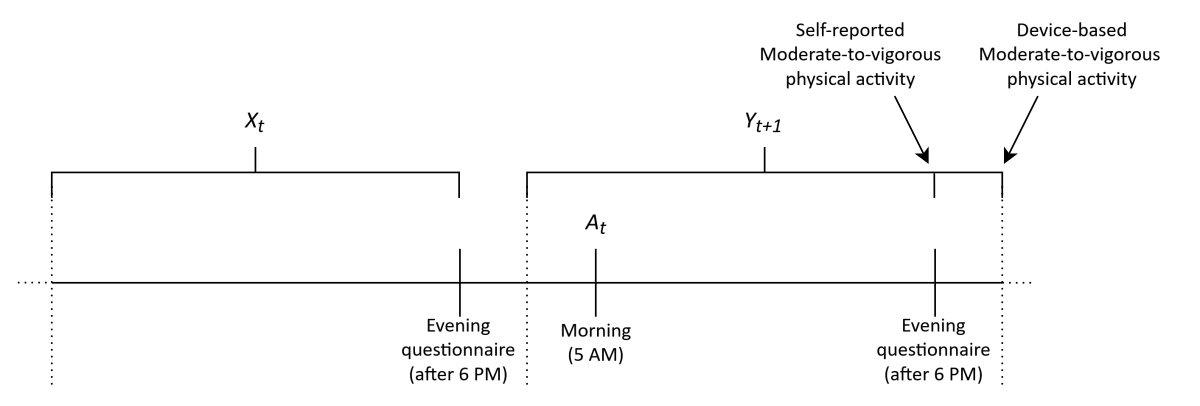


Finally, the dyadic JITAIs in the evening were based on the responses from the daily questionnaire from the current evening. These intervention options aimed to reflect on the engagement and goal progress and to increase engagement in MVPA and adherence to the plans in the upcoming days. Specifically, they aimed at the day of the next planned activity. Thus, the proximal outcome is on the first day, for which couples had planned an activity (see Figure S4). The Figure shows an example of a dyadic JITAI in the evening. Here the JITAI was triggered in the evening of the day with a planned activity (A_t_) based on the information from the daily questionnaires from the same day (X_t_). The proximal outcome is the level of MVPA on the day of the next planned activity (Y_t+1_). Note that the level of MVPA on days without planned activity is no proximal outcome.

**Figure S4.** Implementation of the dyadic JITIAs in the evening.


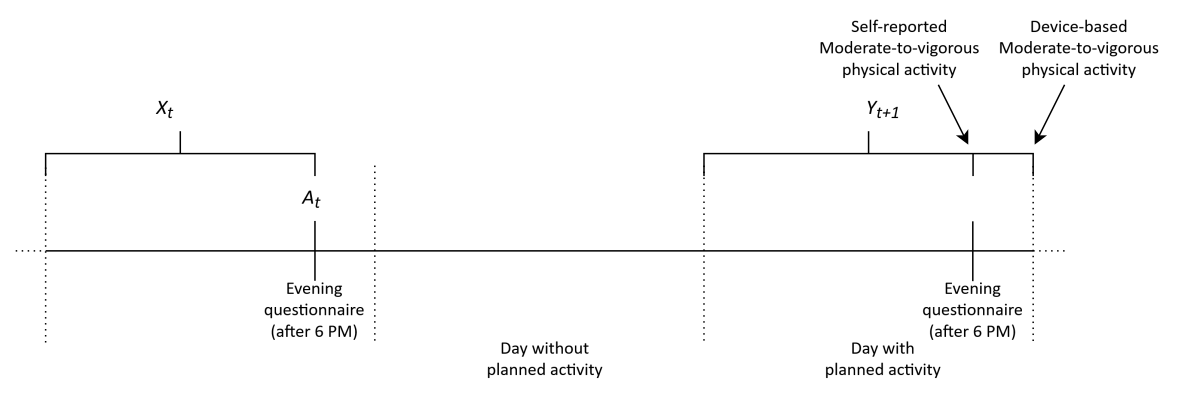


## **Social Exchange Processes**

The dyadic JITAIs targeted various social exchange processes (see Table S3). Different social exchange processes may be targeted depending on the couple’s situation. A list of all intervention options can be found on OSF.

**Table S3.** List of all social exchange processes targeted by the dyadic JITAIs.

| Broad social exchange process | Specific social exchange process | Example of a dyadic JITAIs^a^ |
| --- | --- | --- |
|  |  |  |
| Social support: Providing psychological, material or informational resources intended to benefit the ability to cope with stressors, solve problems, and pursue life opportunities [5,6]. | Emotional support (general): Trying to increase the emotional well-being of the recipient by covering aspects such as encouragement and comfort [7]. | At the beginning of the study, you received information on helpful support. The importance of emotional support was pointed out. This type of support involves creating positive or reducing negative feelings in the other person. Through emotional support, you can help your partner set an appropriate goal during planning and plan enough activities. For example, you can encourage your partner to set a challenging goal during planning, listen to them, or simply show that you are there for them. The important thing is to tailor your support to your partner’s needs. |
|  | Emotional support (joy): Trying to make the planned physical activity more fun for the partner. | Please try to support your partner emotionally (e.g., encourage, listen, give positive feedback, etc.) to be physically active. |
|  | Emotional support (comforting): Trying to comfort the partner after failing to engage in a planned activity. | If your partner did not manage to be physically active today as desired, try to support them emotionally today (e.g., cheer them up, comfort them, put it in perspective, etc.). Also, tell them that you believe that they can manage to be physically active in the future. |
|  | Emotional support (reassurance): Trying to reassure the partner that it is alright to not engage in the activity as planned. | If your partner does not want to set an ambitious goal and plan sufficient activities today during the planning for good reasons, please reassure them that this is okay (e.g. accept, relativise, etc.). Also, let them know that you believe that it is possible to set an ambitious goal and plan enough activities. |
|  | Practical support: Trying to support the partner with practical help, such as giving advice or assistance, involves providing the necessary resources and materials for someone to engage in physical activity, such as gym memberships and exercise equipment [7]. | Please try to provide practical support to your partner today (e.g., do something to help, like going shopping, etc.) to enable them to be physically active. |
|  | Promoting self-efficacy: Trying to convince the partner that they can perform the planned activity and/or increase their level of physical activity. | Show your partner that you believe they can manage to be physically active. |
| Social control: Deliberately and intentionally attempting to change what another person thinks, feels, or does towards an outcome desired by the agent of change [8]. | Negative social control: Trying to influence the partner’s physical activity by autonomy-limiting strategies, such as coercion, eliciting negative emotions, or withdrawal [9,10]. | At the beginning of the study, you received information on helpful support. The importance of motivation was emphasised. The intention here is to influence the behaviour of the other person in a certain direction. However, how this is approached is crucial. There are strategies that are well suited to this and those that are less appropriate. Below is a list of some strategies that are well and less well suited to achieving the desired goals:  Strategies that work well:   - Demonstrate the positive consequences of physical activity - Emphasise the importance of physical activity - Make suggestions   Strategies that are less suitable:   - Threatening - Blaming - Ignoring the other person   These two types of strategies have different effects. The upper strategies can help the other person to increase their physical activity. With the lower strategies, there is a greater risk that the other person will do exactly the opposite of what you want. In addition, the upper strategies can have a positive effect on your relationship, while the lower strategies can have a negative effect on the quality of your relationship. |
|  | Positive social control: Trying to influence the partner’s physical activity by autonomy-supporting strategies, such as persuasion or discussions [9,10]. | At the beginning of the study, you received information on helpful support. The importance of motivating was pointed out. This is about influencing the other person’s behaviour in a certain direction. In this way, you can persuade your partner to do the activity you have planned. For example, you can show your partner the importance and benefits of physical activity. |
|  | Convincing about the advantages: Trying to convince the partner about the advantages or refuting the disadvantages of physical activity. | Over the next few days, please try to convince your partner to be physically active by pointing out the advantages or refuting the disadvantages of physical activity. |
|  | Building commitment: Trying to increase the partner’s perceived importance to reach the goal. | Please discuss together with your partner, the reasons why you want to increase your physical activity. |
|  | Emphasise commitment: State the importance of your joint commitment. A person can only emphasise commitment if they have high commitment. | Please point out to your partner today that you have a joint goal of being more physically active. Also, emphasise that you want to pursue this goal together, even if it takes great effort or if obstacles get in your way. |
| Sabotaging: Actively trying to reduce engagement in the health behaviour of the partner [11]. | Reduce sabotaging: Stopping the act of sabotaging. | Please think about whether your partner has tried to influence you to be (not or not very) physically active in the past few days by nagging, blaming, or other negative strategies. If this is the case, ask them to avoid this in the future. |
| Action planning: Planning when, where and how to engage in the behaviour [4].  Coping planning: Anticipating personal risk situations (i.e. situations that endanger the performance of intended behaviour) and planning coping responses in detail [4]. | Dyadic planning: Creating plans together with a partner on when, where, and how one partner will implement a behaviour [12]. | During today’s planning, ask your partner if they would like to adjust the goal or activities you have set and if so, what activities they would like to plan. If you also like the suggested changes, you can include them in your planning. |
|  | Collaborative planning: *C*reating plans together with a partner on when, where, and how both partners will implement a behaviour together [13] |  |

^a^The wordings of the dyadic just-in-time adaptive interventions are rough translations from German.

## **Dyadic Intervention Techniques**

We categorised the dyadic JITAIs used in our study according to the compendium of dyadic intervention techniques (DITs) developed by Di Maio et al. [14]. According to this compendium, DITs can be coded based on their execution level, their intervention task, and their target level. The execution level describes who completes the intervention task (i.e., alone by one dyad member or together by the dyad). The intervention task describes the task itself that is prompted by the intervention option. The target level defines who is targeted by the content of the intervention task (i.e., one dyad member or the dyad). Finally, we added the theoretical determinants of the DITs according to the theoretical domains framework [15]. The categorisation of all dyadic JITAIs aiming to increase MVPA used in the current study can be found on OSF.

## **Descriptive Statistics of the JITAIs**

We calculated the frequency and distribution of various types of dyadic JITAIs (see Table S4). Participants responded to dyadic JITAIs 74.3% of the time they were sent.

**Table S4**. Descriptive statistics of the JITAIs.^a^

| Parameter | *M* | *SD* | Min | Max | Skewness | Kurtosis |
| --- | --- | --- | --- | --- | --- | --- |
|  |  |  |  |  |  |  |
| JITAIs per week | 2.64 | 1.26 | 0.33 | 6.00 | 0.47 | −0.33 |
| Cross-over JITAIs per week | 1.57 | 0.99 | 0.00 | 4.67 | 0.86 | 0.44 |
| Joint JITAIs per week | 1.07 | 0.78 | 0.00 | 3.00 | 0.58 | −0.30 |
| JITAIs before planning per week | 1.14 | 0.96 | 0.00 | 4.00 | 1.09 | 0.99 |
| JITAIs before planned activity per week | 0.31 | 0.22 | 0.00 | 1.00 | 0.51 | 0.32 |
| JITAIs in the evening per week | 1.20 | 0.56 | 0.00 | 2.33 | 0.30 | -0.36 |

^a^Frequencies of the dyadic JITAIs during the intervention phase.

## **References**

1. Nahum-Shani I, Smith SN, Spring BJ, Collins LM, Witkiewitz K, Tewari A, Murphy SA. Just-in-time adaptive interventions (JITAIs) in mobile health: Key components and design principles for ongoing health behavior support. Annals of Behavioral Medicine 2018 May 18;52(6):446–462. doi: 10.1007/s12160-016-9830-8

2. Qian T, Walton AE, Collins LM, Klasnja P, Lanza ST, Nahum-Shani I, Rabbi M, Russell MA, Walton MA, Yoo H, Murphy SA. The microrandomized trial for developing digital interventions: Experimental design and data analysis considerations. Psychological Methods 2022 Jan 13; doi: 10.1037/met0000283

3. Boruvka A, Almirall D, Witkiewitz K, Murphy SA. Assessing Time-Varying Causal Effect Moderation in Mobile Health. Journal of the American Statistical Association 2018 Jul 3;113(523):1112–1121. doi: 10.1080/01621459.2017.1305274

4. Sniehotta FF, Schwarzer R, Scholz U, Schüz B. Action planning and coping planning for long-term lifestyle change: theory and assessment. Eur J Soc Psychol 2005 Jul;35(4):565–576. doi: 10.1002/ejsp.258

5. Cohen S. Social relationships and health. American Psychologist 2004;59(8):676–684.

6. Feeney BC, Collins NL. A New Look at Social Support: A Theoretical Perspective on Thriving Through Relationships. Pers Soc Psychol Rev 2015 May;19(2):113–147. doi: 10.1177/1088868314544222

7. Schwarzer R, Knoll N. Social support. Health psychology 2nd ed Great Britain: Blackwell: Osford; 2010. p. 283--293. Available from: https://www.scopus.com/record/display.uri?eid=2-s2.0-84856653408&origin=inward

8. Craddock E, vanDellen MR, Novak SA, Ranby KW. Influence in Relationships: A Meta-Analysis on Health-Related Social Control. Basic and Applied Social Psychology 2015 Mar 4;37(2):118–130. doi: 10.1080/01973533.2015.1011271

9. Scholz U, Stadler G, Berli C, Lüscher J, Knoll N. How do people experience and respond to social control from their partner? Three daily diary studies. Front Psychol 2021 Jan 13;11:613546. doi: 10.3389/fpsyg.2020.613546

10. Lewis MA, Butterfield RM. Antecedents and Reactions to Health-Related Social Control. Pers Soc Psychol Bull 2005 Mar;31(3):416–427. doi: 10.1177/0146167204271600

11. Ogden J, Quirke-McFarlane S. Sabotage, Collusion, and Being a Feeder: Towards a New Model of Negative Social Support and Its Impact on Weight Management. Curr Obes Rep 2023 Jun 7;12(2):183–190. doi: 10.1007/s13679-023-00504-5

12. Burkert S, Scholz U, Gralla O, Roigas J, Knoll N. Dyadic planning of health-behavior change after prostatectomy: A randomized-controlled planning intervention. Social Science & Medicine 2011 Sep;73(5):783–792. doi: 10.1016/j.socscimed.2011.06.016

13. Prestwich A, Conner M, Lawton R, Bailey W, Litman J, Molyneaux V. Individual and collaborative implementation intentions and the promotion of breast self-examination. Psychology & Health 2005 Dec;20(6):743–760. doi: 10.1080/14768320500183335

14. Di Maio S, Villinger K, Knoll N, Scholz U, Stadler G, Gawrilow C, Berli C. Compendium of dyadic intervention techniques (DITs) to change health behaviours: a systematic review. Health Psychology Review 2024 Mar 4;1–36. doi: 10.1080/17437199.2024.2307534

15. Atkins L, Francis J, Islam R, O’Connor D, Patey A, Ivers N, Foy R, Duncan EM, Colquhoun H, Grimshaw JM, Lawton R, Michie S. A guide to using the Theoretical Domains Framework of behaviour change to investigate implementation problems. Implementation Sci 2017 Dec;12(1):77. doi: 10.1186/s13012-017-0605-9
