# Supplementary material for: Effectiveness of Smartphone-Based Dyadic Interventions to Increase Physical Activity in Romantic Couples: Microrandomized Trial
Source: JMIR Mhealth Uhealth. 2026 Jan 27;14:e67136. doi: 10.2196/67136 (PMC12892032; doi:10.2196/67136)
Supplement: Multimedia Appendix 5 [file mhealth_v14i1e67136_app5.docx]

**Table S1.** Sensitivity analysis of the effects of the intervention phase on device-based physical activity.^a-c^

|  |  |  |  |  | CI_90_ | |
| --- | --- | --- | --- | --- | --- | --- |
| Fixed effects | Estimate | *SE* | *t* | *P* | Lower | Upper |
|  |  |  |  |  |  |  |
| Intercept | 106.00 | 4.39 | 24.15 | <.001 | 98.78 | 113.22 |
| Intervention phase^d^ | 6.46 | 3.35 | 1.93 | .03 | 0.95 | 11.97 |
| Time^e^ | −0.04 | 0.90 | −0.04 | .97 | −1.53 | 1.45 |
| Weekend^f^ | 12.48 | 1.85 | 6.75 | <.001 | 9.43 | 15.52 |
| Group B^g^ | −3.91 | 8.33 | −0.47 | .64 | −17.99 | 10.17 |
| Group C^g^ | 6.87 | 8.93 | 0.77 | .45 | −8.22 | 21.96 |
| Skilled support^h^ | −7.45 | 3.94 | −1.89 | .06 | −13.94 | −0.96 |
| Barriers^i^ | 0.70 | 0.15 | 4.65 | <.001 | 0.45 | 0.95 |
| Facilitating factors_j_ | 1.26 | 0.14 | 8.87 | <.001 | 1.03 | 1.50 |
| Wear time^k^ | 0.15 | 0.01 | 13.62 | <.001 | 0.13 | 0.17 |
|  |  |  |  |  | CI_90_ | |
| Random effects | Estimate | *SE* | *z* | *P* | Lower | Upper |
|  |  |  |  |  |  |  |
| Level-2 |  |  |  |  |  |  |
| Intercept | 23.84 | 4.53 | 5.27 | <.001 | 17.53 | 32.42 |
| Intervention phase | 8.65 | 3.62 | 2.39 | .02 | 4.55 | 16.47 |
| Day | 4.10 | 0.88 | 4.66 | <.001 | 2.90 | 5.80 |
| Wear time | 0.06 | 0.01 | 5.75 | <.001 | 0.04 | 0.08 |
| Level-1 |  |  |  |  |  |  |
| Residuals | 49.11 | 0.65 | 75.84 | <.001 | 48.06 | 50.19 |
| Autocorrelation | 0.29 | 0.02 | 16.53 | <.001 | 0.26 | 0.31 |

^a^All covariates were grand-mean centred.

^b^All *p*-values are two-tailed except those of the intervention phase, where one-tailed *p*-values are used.

^c^Number of couples = 38; number of days = 55. Number of cases in the analysis = 3706.

^d^Intervention phase (coded as 1) included all days during the period when the couple received dyadic interventions and was compared to the control phase (coded as 0).

^e^Time was grand-mean-centred per 7 days.

^f^Weekend included Saturday and Sunday (coded as 1), and weekday included the remaining days (coded as 0).

^g^Dummy variables indicating if the participants were in intervention group B or group C (coded as 1) or not (coded as 0). Group A serves as the reference group which was in the study phase from week two to eight. The study phase for group B took place from week two to four. The study phase for group C took place from week six to eight.

^h^Dummy variable indicating if the couple received the skilled support intervention (coded as 1) or has not yet received the skilled support intervention (coded as 0).

^i^Sum scores of all barriers.

^j^Sum scores of all facilitating factors.

^k^Wear time of the accelerometers in minutes.

**Table S2.** Sensitivity analysis of the effects of the intervention phase on self-reported physical activity. ^a-c^

|  |  |  |  |  | CI_90_ | |
| --- | --- | --- | --- | --- | --- | --- |
| Fixed effects | Estimate | *SE* | *t* | *P* | Lower | Upper |
|  |  |  |  |  |  |  |
| Intercept | 24.32 | 2.52 | 9.66 | <.001 | 20.18 | 28.47 |
| Intervention phase^d^ | 8.75 | 3.63 | 2.41 | .008 | 2.77 | 14.72 |
| Time^e^ | 0.07 | 0.85 | 0.08 | .94 | −1.33 | 1.46 |
| Weekend^f^ | 7.16 | 1.64 | 4.36 | <.001 | 4.46 | 9.86 |
| Group B^g^ | 2.45 | 5.93 | 0.41 | .68 | −7.58 | 12.47 |
| Group C^g^ | 8.77 | 6.29 | 1.40 | .17 | −1.85 | 19.40 |
| Skilled support^h^ | −15.13 | 3.28 | −4.62 | <.001 | −20.52 | −9.74 |
| Barriers^i^ | 1.69 | 0.14 | 12.01 | <.001 | 1.46 | 1.92 |
| Facilitating factors^j^ | 2.74 | 0.13 | 20.69 | <.001 | 2.53 | 2.96 |
|  |  |  |  |  | CI_90_ | |
| Random effects | Estimate | *SE* | *z* | *P* | Lower | Upper |
|  |  |  |  |  |  |  |
| Level-2 |  |  |  |  |  |  |
| Intercept | 11.63 | 1.87 | 6.21 | <.001 | 8.95 | 15.11 |
| Intervention phase | 16.50 | 2.75 | 5.99 | <.001 | 12.58 | 21.65 |
| Time | 4.19 | 0.62 | 6.72 | <.001 | 3.29 | 5.34 |
| Level-1 |  |  |  |  |  |  |
| Residuals | 45.81 | 0.53 | 86.92 | <.001 | 44.95 | 46.68 |
| Autocorrelation | 0.05 | 0.02 | 2.81 | .005 | 0.02 | 0.07 |

^a^All covariates were grand-mean centred.

^b^All *p*-values are two-tailed except those of the intervention phase, where one-tailed *p*-values are used.

^c^Number of couples = 38; number of days = 55. Number of cases in the analysis = 3944.

^d^Intervention phase (coded as 1) included all days during the period when the couple received dyadic interventions and was compared to the control phase (coded as 0).

^e^Time was grand-mean-centred per 7 days.

^f^Weekend included Saturday and Sunday (coded as 1), and weekday included the remaining days (coded as 0).

^g^Dummy variables indicating if the participants were in intervention group B or group C (coded as 1) or not (coded as 0). Group A serves as the reference group which was in the study phase from week two to eight. The study phase for group B took place from week two to four. The study phase for group C took place from week six to eight.

^h^Dummy variable indicating if the couple received the skilled support intervention (coded as 1) or has not yet received the skilled support intervention (coded as 0).

^i^Sum scores of all barriers.

^j^Sum scores of all facilitating factors.

**Table S3.** Sensitivity analysis of the effects of the planning interventions and dyadic JITAIs on device-based and self-reported MVPA.^a-c^

|  | Device-based physical activity | | | | Self-reported physical activity | | | |
| --- | --- | --- | --- | --- | --- | --- | --- | --- |
| Parameter | Estimate | *P* | CI_90_ | | Estimate | *P* | CI_90_ | |
|  |  |  | Lower | Upper |  |  | Lower | Upper |
|  |  |  |  |  |  |  |  |  |
| Intercept | 107.74 | <.001 | 100.97 | 114.52 | 29.53 | <.001 | 25.25 | 33.82 |
| Planning^d^ | −0.40 | .46 | −7.35 | 6.54 | 0.14 | .48 | −5.52 | 5.80 |
| JITAI_Actor_^e^ | 5.59 | .049 | 0.04 | 11.13 | 2.20 | .25 | −3.29 | 7.69 |
| JITAI_Partner_^f^ | 6.65 | .03 | 1.11 | 12.20 | 8.39 | .01 | 2.26 | 14.53 |
| Time^g^ | −0.88 | .27 | −2.23 | 0.46 | −0.58 | .52 | −2.06 | 0.91 |
| Weekend^h^ | 14.41 | <.001 | 8.82 | 20.01 | 8.22 | .002 | 4.22 | 12.21 |
| Group B^i^ | −0.05 | 1.00 | −15.81 | 15.72 | 4.17 | .54 | −7.22 | 15.55 |
| Group C^i^ | 2.41 | .83 | −16.65 | 21.47 | 8.68 | .10 | −0.09 | 17.45 |
| Skilled support^j^ | −3.19 | .49 | −10.89 | 4.50 | −10.01 | .09 | −19.58 | −0.44 |
| Barriers^k^ | 0.53 | .15 | −0.07 | 1.14 | 1.59 | <.001 | 0.93 | 2.24 |
| Facilitating factors^l^ | 1.28 | .009 | 0.51 | 2.06 | 2.92 | <.001 | 2.18 | 3.66 |
| Wear time^m^ | 0.15 | <.001 | 0.13 | 0.18 |  |  |  |  |

^a^All predictor variables were grand-mean centred.

^b^All *p*-values are two-tailed except those of the intervention phase, where one-tailed *p*-values are used.

^c^Number of couples = 38; number of days = 55. Number of cases in the analysis for device-based MVPA = 3706 and for self-reported MVPA = 3944.

^d^Days with planned physical activities (coded as 1) were compared to days without any planned physical activities (coded as 0).

^e^Days on which a dyadic JITAI targeted the actor’s MVPA (coded as 1) were compared to days without dyadic JITAIs targeting the actor’s MVPA (coded as 0).

^f^Days on which a dyadic JITAI targeted the partner’s MVPA (coded as 1) were compared to days without dyadic JITAIs targeting the partner’s MVPA (coded as 0).

^g^Time was grand-mean-centred per 7 days.

^h^Weekend included Saturday and Sunday (coded as 1), and weekday included the remaining days (coded as 0).

^i^Dummy variables indicating if the participants were in intervention group B or group C (coded as 1) or not (coded as 0). Group A serves as the reference group which was in the study phase from week two to eight. The study phase for group B took place from week two to four. The study phase for group C took place from week six to eight.

^j^Dummy variable indicating if the couple received the skilled support intervention (coded as 1) or has not yet received the skilled support intervention (coded as 0).

^k^Sum scores of all barriers.

^l^Sum scores of all facilitating factors.

^m^Wear time of the accelerometers in minutes.

**Table S4.** Sensitivity analysis of the effects of the dyadic JITAIs on device-based and self-reported MVPA without barriers and facilitating factors.^a-c^

|  | Device-based physical activity | | | | Self-reported physical activity | | | |
| --- | --- | --- | --- | --- | --- | --- | --- | --- |
| Parameter | Estimate | *P* | CI_90_ | | Estimate | *P* | CI_90_ | |
|  |  |  | Lower | Upper |  |  | Lower | Upper |
|  |  |  |  |  |  |  |  |  |
| Intercept | 107.65 | <.001 | 100.75 | 114.55 | 30.27 | <.001 | 24.36 | 36.18 |
| Planning^d^ | 3.17 | .25 | −4.65 | 10.99 | 10.19 | .010 | 3.17 | 17.20 |
| JITAI_Actor_^e^ | 12.59 | <.001 | 7.38 | 17.80 | 18.68 | <.001 | 11.94 | 25.42 |
| JITAI_Partner_^f^ | 8.80 | .01 | 2.55 | 15.05 | 13.79 | .002 | 6.28 | 21.31 |
| Time^g^ | −0.94 | .25 | −2.33 | 0.44 | −0.81 | .43 | −2.53 | 0.91 |
| Weekend^h^ | 15.50 | <.001 | 9.68 | 21.33 | 10.41 | <.001 | 6.16 | 14.67 |
| Group B^i^ | 0.55 | .95 | −15.69 | 16.79 | 5.73 | .42 | −6.06 | 17.52 |
| Group C^i^ | 7.45 | .52 | −11.89 | 26.80 | 20.42 | .04 | 4.02 | 36.82 |
| Skilled support^j^ | −6.28 | .14 | −13.38 | 0.82 | −17.87 | .005 | −27.74 | −7.99 |
| Wear time^k^ | 0.16 | <.001 | 0.14 | 0.18 |  |  |  |  |

^a^All predictor variables were grand-mean centred.

^b^All *p*-values are two-tailed except those of the intervention phase, where one-tailed *p*-values are used.

^c^Number of couples = 38; number of days = 55. Number of cases in the analysis for device-based MVPA = 3706 and for self-reported MVPA = 3944.

^d^Days with planned physical activities (coded as 1) were compared to days without any planned physical activities (coded as 0).

^e^Days on which a dyadic JITAI targeted the actor’s MVPA (coded as 1) were compared to days without dyadic JITAIs targeting the actor’s MVPA (coded as 0).

^f^Days on which a dyadic JITAI targeted the partner’s MVPA (coded as 1) were compared to days without dyadic JITAIs targeting the partner’s MVPA (coded as 0).

^g^Time was grand-mean-centred per 7 days.

^h^Weekend included Saturday and Sunday (coded as 1), and weekday included the remaining days (coded as 0).

^i^Dummy variables indicating if the participants were in intervention group B or group C (coded as 1) or not (coded as 0). Group A serves as the reference group which was in the study phase from week two to eight. The study phase for group B took place from week two to four. The study phase for group C took place from week six to eight.

^j^Dummy variable indicating if the couple received the skilled support intervention (coded as 1) or has not yet received the skilled support intervention (coded as 0).

^k^Wear time of the accelerometers in minutes.
